# Phasic dopamine reinforces distinct striatal stimulus encoding in the olfactory tubercle driving dopaminergic reward prediction

**DOI:** 10.1038/s41467-020-17257-7

**Published:** 2020-07-10

**Authors:** Lars-Lennart Oettl, Max Scheller, Carla Filosa, Sebastian Wieland, Franziska Haag, Cathrin Loeb, Daniel Durstewitz, Roman Shusterman, Eleonora Russo, Wolfgang Kelsch

**Affiliations:** 1 Department of Psychiatry and Psychotherapy, Central Institute of Mental Health, Medical Faculty Mannheim, Heidelberg University, 68159 Mannheim, Germany; 2 Department of Psychiatry and Psychotherapy, University Medical Center, Johannes Gutenberg University, 55131 Mainz, Germany; 3 Department of Theoretical Neuroscience, Central Institute of Mental Health, Medical Faculty Mannheim, Heidelberg University, 68159 Mannheim, Germany; 4 Institute of Neuroscience, University of Oregon, Eugene, OR 97403 USA; 5 Present Address: Sainsbury Wellcome Centre for Neural Circuits and Behaviour, London, W1T 4JG UK

**Keywords:** Neural encoding, Classical conditioning, Neural circuits, Olfactory cortex, Reward

## Abstract

The learning of stimulus-outcome associations allows for predictions about the environment. Ventral striatum and dopaminergic midbrain neurons form a larger network for generating reward prediction signals from sensory cues. Yet, the network plasticity mechanisms to generate predictive signals in these distributed circuits have not been entirely clarified. Also, direct evidence of the underlying interregional assembly formation and information transfer is still missing. Here we show that phasic dopamine is sufficient to reinforce the distinctness of stimulus representations in the ventral striatum even in the absence of reward. Upon such reinforcement, striatal stimulus encoding gives rise to interregional assemblies that drive dopaminergic neurons during stimulus-outcome learning. These assemblies dynamically encode the predicted reward value of conditioned stimuli. Together, our data reveal that ventral striatal and midbrain reward networks form a reinforcing loop to generate reward prediction coding.

## Abstract (web summary)

It is not entirely understood how network plasticity produces the coding of predicted value during stimulus-outcome learning. Here, the authors reveal a reinforcing loop in distributed limbic circuits, transforming sensory stimuli into reward prediction coding broadcasted by dopamine neurons to the brain.

## Introduction

Relevant events in the external world can be predicted through learning of associations between stimuli and outcomes. During stimulus–outcome learning, an initially neutral stimulus becomes a conditioned stimulus (CS+) when paired repeatedly with a reward as unconditioned stimulus (US). Midbrain dopamine neurons (DAN) show transient firing responses to US and CS+^1–6^. These firing responses encode the reward prediction error (RPE) signal, which computes the mismatch between the value of the received reward and the expected reward. These transient firing responses of DAN elicit phasic dopamine release that is thought to provide a reinforcement learning signal to the ventral striatum^1^. RPE signals change with learning^7,8^. Initially, when stimulus–outcome relationships are uncertain, DAN mainly fire bursts at US, reflecting the positive surprise to the obtained rewards^1,9^. Then, during learning, an additional response evolves at CS+, reflecting the reward predicted by the conditioned stimulus^1,9^. This latter evolving signal represents the prediction component of the RPE. Despite broad experimental evidence of this reward prediction signal, the mechanisms leading to its formation are still not entirely clear^1^.

DAN receive inputs from distributed brain networks^10^. In these circuits, stimulus value is dynamically encoded by populations of neurons with excitatory and inhibitory stimulus responses that evolve with learning^11,12^. Reward prediction is represented in the ventral striatum (VS), as shown in human imaging studies and recordings from other species^1,8,13,14^. Neuronal activity in the VS is modified when subjects learn to predict future rewards from sensory cues^1,14,15^. VS and DAN interactions take place in a network of reciprocal direct and indirect connections, resulting in a mutual functional modulation. On the one hand, phasic dopamine release to VS is critical to reinforcement learning^6,16^, induces synaptic plasticity to SPN in vitro^17–19^, and, at the level of behavior, is sufficient to generate conditioned responses to stimuli^20–22^. On the other hand, theoretical and experimental work has suggested that VS is an important source of the reward prediction component to DAN^4,10,14,23–27^. For instance, systematic input mapping^10^ has revealed that VS contains a relatively high fraction of neurons encoding dominantly stimulus-driven expectation coding among the regions that provide input to DAN.

The emergence of the reward prediction signals during learning should, therefore, result from the reciprocal interactions between VS and VTA. Within this emerging concept, direct experimental evidence is missing for two important points: It is not entirely clear whether phasic dopamine (pDA) release is sufficient to induce plasticity in the encoding of stimuli in VS of awake animals and through which network modifications this plasticity is achieved. Moreover, direct evidence of the coordinated inter-areal activity is missing for the directional information transfer mediating the learned stimulus–outcome association from striatal projection neurons (SPN) to DAN.

VS is composed of the olfactory tubercle (OTu) and nucleus accumbens. The OTu provides the main direct access of olfactory information and also input from other sensory modalities to the limbic reward system^28,29^ and shares functions in stimulus–outcome learning with nucleus accumbens^22,28,30^. The OTu of VS is therefore of particular interest to understand the interaction of striatal networks with VTA in generating reward prediction signals from sensory cues. We tested in awake animals first whether, in the absence of physical rewards, optogenetically evoked pDA release is sufficient to induce selective plasticity in SPN of the OTu. Second, we tested whether, during the formation of stimulus–outcome associations, the evolving CS+ representations in OTu may generate a prediction signal that leads DAN firing. For this latter aim, we examined the dynamics of interregional assemblies from dual-site VS–VTA recordings of a within-session reversal learning task.

## Results

### Phasic dopamine induces plasticity to striatal odor responses

In behavioral tasks, it is difficult to isolate the direct effects of DA on the neuronal representation of stimuli. First, it cannot be disentangled whether changes in stimulus encoding are determined by direct effects of DA on the striatal network or rather by task-related motivational states and preparation for reward retrieval. Second, the manipulation of DA release changes behavior. Behavioral changes impact neuronal coding. It is therefore difficult in tasks involving animal behavior to disentangle indirect DA effects from those directly induced by DA in the target circuit. To examine the effects of pDA on sensory representations in the ventral striatal OTu, we designed a paradigm where pDA release is decoupled from reward retrieval in awake mice.

**Fig. 1:**
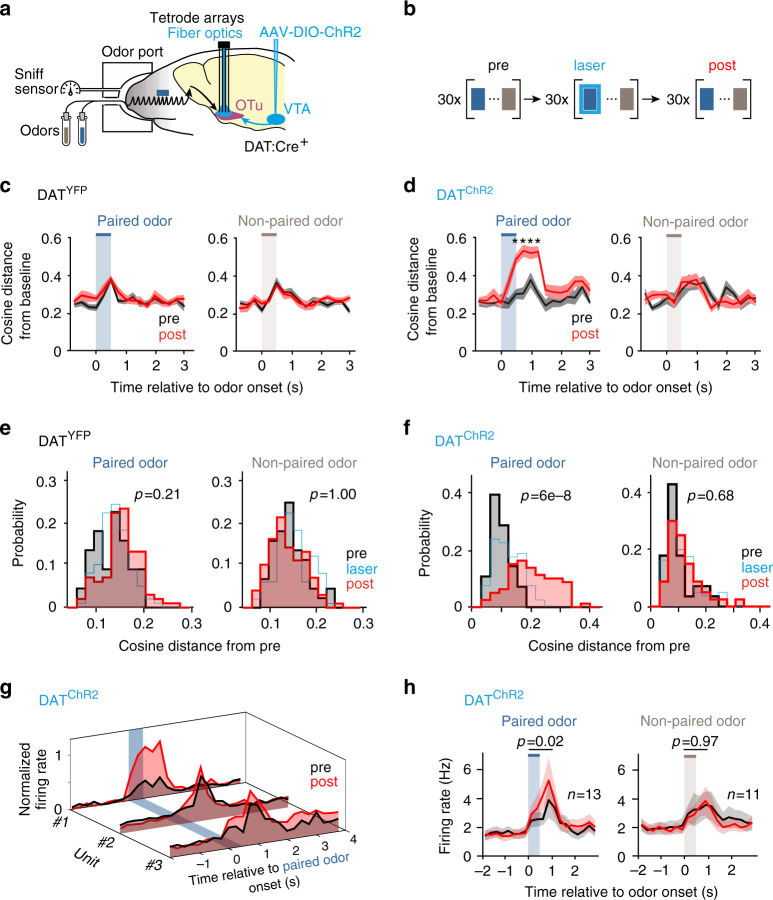
Phasic DA modifies striatal population encoding selectively of the paired odor. **a** Two odors were applied for 0.5 s in pseudorandomized order to head-fixed DAT^ChR2^ or DAT^YFP^ mice. **b** During the ‘pairing’ phase, one odor was paired transiently with brief laser trains delivered to the recording site in the OTu to evoke phasic DA (pDA) release. **c**, **d** Cosine distance from baseline of the of the population vector for the two odors during the ‘pre’ and ‘post’ phases (displayed mean ± S.E.). Phasic DA pairing enhanced exclusively the paired odor response of (**d**) DAT^ChR2^, but not (**c**) DAT^YFP^ mice (two-sided *t*-test, asterisks mark significance at *α* = 0.05 with Benjamini–Hochberg correction). DAT^YFP^: *n* = 10 trial-averages of three trials, respectively, for both ‘pre’ and ‘post’; DAT^ChR2^: *n* = 8/10 trial-averages of three trials, respectively, for ‘pre’/‘post’. **e**, **f** Distribution of cosine distances between response vectors within the ‘pre’ phase (black) and between the ‘pre’ and ‘post’ phase (red). Only the response to the paired odor of (**f**) DAT^ChR2^ mice changed after pDA pairing (three-way ANOVA; factors: cohort, phase, odor; interaction effect: F(1,498) = 8.0, *p* = 0.005; post hoc tests indicated, Tukey’s correction). **g** Example of the normalized peri-stimulus histograms (PSTH) for excitatory odor responses of 3 SPN in DAT^ChR2^ mice for ‘pre’ and ‘post’ phases. **h** Mean PSTH ± S.E. of SPN with excitatory responses to the paired (left) and non-paired (right) odor in DAT^ChR2^ mice (two-sided paired Wilcoxon signed-rank test of the averaged time bin from 0 to 1 s) (see also Supplementary Fig. 3e,f). Source data are provided as a Source data file. See also Supplementary Figs. 1–3.

We recorded single units from the OTu (Fig. 1a, Supplementary Fig. 1a-b) with a custom-designed light-weight tetrode array that was chronically implanted and allowed for recordings with up to 128 channels (Supplementary Fig. 1c-f). Sniffing activity was continuously monitored (Fig. 1a). Release of pDA was evoked through fiber optics placed bilaterally above OTu in DAT:Cre mice that were injected with a Cre-dependent AAV and expressed ChR2 selectively in DAN (DAT^ChR2^) (Supplementary Fig. 2a). Animals with selective expression of YFP in DAN served as a control cohort (DAT^YFP^). DAT^YFP^ mice were also implanted with optic fibers above OTu to control for intracranial heating effects.

We designed a protocol consisting of three phases (Fig. 1b). Brief 0.5 s bouts of two natural flower odors were applied throughout all phases in a pseudorandomized order. After sampling odor responses in a ‘pre’ phase, one of the two odors was paired with a brief burst of optogenetic excitation of dopaminergic terminals in the ventral striatal OTu (12 pulses at 40 Hz) (‘pairing’ phase); then a ‘post’ phase followed to track the further evolution of odor responses after evoked pDA. We will refer to these two odors as ‘paired’ and ‘non-paired’ odors, respectively. Except for the pairing phase of the paired odor, all odor presentations were combined with a sham laser light of the same wavelength and intensities above the animals’ head to account for visual stimulation effects (12 pulses at 40 Hz). It is important to note that mice never experienced reward following stimulus presentation, neither prior to the experiment, nor during it.

We recorded a total of 195 single units in 6 DAT^YFP^ mice and 198 units in 6 DAT^ChR2^ mice. Among them, only units with a baseline firing rate <5 Hz were considered SPN (Supplementary Fig. 2b). We analyzed only units with stable odor responses throughout the ‘pre’ phase. SPN displayed inhibitory or excitatory responses to the odors. The time to peak odor response varied among SPN between 0 to 1 s (Supplementary Fig. 2c).

To examine pDA-induced plasticity, we computed the distance from baseline of the population vector during the ‘pre’ and ‘post’ phases. Distances were computed both with a cosine and a Euclidean metric to account for differences in angle and rate in the population activity. This approach is sensitive to small changes in rate, if coherent throughout the population, and sums the contribution of both excitatory and inhibitory responses. In DAT^YFP^ mice, no change was observed from ‘pre’ to ‘post’ for either odor response (Fig. 1c, Supplementary Fig. 2d). In DAT^ChR2^ mice, the deflection from baseline increased selectively in response to the paired odor; while it remained unchanged for the non-paired odor (Fig. 1d, Supplementary Fig. 2d). Thus, the enhanced deflection from baseline corresponded to a selective change in SPN responses to the paired stimulus. In fact, the change in odor response from the ‘pre’ to ‘post’ phase exceeded the trial-by-trial variability within the ‘pre’ phase only for the paired odor in DAT^ChR2^ mice (Fig. 1e, f, Supplementary Fig. 2e). The selective plasticity of the paired odor response was also confirmed when neuronal activity was aligned to the onset of the first inhalation after stimulus onset (Supplementary Fig. 2f). SPN displayed relatively long firing rate increases over multiple sniff cycles to even short odor presentations (cf. Fig. 1). These net firing rate increases were not correlated to the sniff frequency in DAT^YFP^ mice (data not shown). Further, population vectors were obtained by concatenating units across sessions with maintained trial order. We excluded that the specific order of trial concatenation across sessions influenced our results by repeating all main analyses with 300 random permutations of cross-session trial matching (see Supplementary Material and Supplementary Fig. 3a-d).

At the single-unit level, we observed plasticity only for excitatory responses in DAT^ChR2^ mice (Fig. 1g, h). After the pairing phase, this subset increased its firing response to the paired odor, while no change occurred in response to the non-paired odor. Inhibitory stimulus responses remained unchanged in DAT^ChR2^ mice and no changes were observed for responses in DAT^YFP^ mice (Supplementary Fig. 3e,f). The effects of pDA stimulation on population and single-unit odor responses persisted without further stimulation for at least 20 min (‘post’ phase). In summary, pDA evoked plasticity selectively in the subpopulation with excitatory response to paired sensory objects.

### Phasic dopamine increases discriminability of odor encoding

**Fig. 2:**
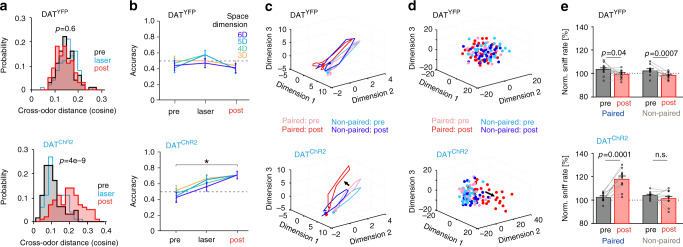
Phasic DA increases the difference between the paired and non-paired odor encoding and improves decoding. **a** Distribution of the cosine distance between the two odor responses during ‘pre’ and ‘post’ phases. After pDA pairing, cross-odor distance increased only in DAT^ChR2^ mice (two-way ANOVA; factors: cohort, odor; interaction effect: F(1,360) = 90.6, *p* = 3 × 10^−19^; post hoc comparisons indicated, Tukey’s correction). **b** Quadratic discriminant analysis between paired and non-paired odor responses. Only in DAT^ChR2^ mice, laser pairing improved the average accuracy (two-sided Fisher’s exact test between ‘pre’ vs ‘post’; multiple state space dimensions are tested to confirm the robustness, *α* = 0.05 with Benjamini–Hochberg correction on tests performed on all dimensions and cohorts, DAT^ChR2^: asterisk mark significance for all dimensions, DAT^YFP^: no significance in any dimension. DAT^YFP^: *n* = 60 accuracy values for all dimensions and phases; DAT^ChR2^: *n* = 52/62/62 accuracy values for all dimensions and for ‘pre’/’laser’/’post’ phases, resp.; displayed mean ± S.E.). **c** Averaged trajectories for paired and non-paired SPN odor responses visualized through factor analysis after time embedding (dots mark the beginning of the trial). Trajectories were jointly rotated to improve visualization. **d** Trial-specific odor responses visualized through multidimensional scaling. pDA pairing separated the paired odor representation from its original representation and from the one of the non-paired odor. **e** Mean sniff rate ± S.E. following odor onset normalized to baseline. The sniff rate increased only for the odor paired with pDA stimulation in DAT^ChR2^ mice (two-sided paired *t*-test). Source data are provided as a Source data file. See also Supplementary Figs. 4 and 5.

Such pDA-induced plasticity may render the representation of the paired odor more distinct from other stimuli. Indeed, the distance between the two odor representations increased after pairing in DAT^ChR2^, but not in DAT^YFP^ mice (Fig. 2a, Supplementary Fig. 4). To explicitly test whether this effect enhanced discriminability between the two odor representations, we performed a quadratic discriminant analysis on the population vectors of the paired and non-paired odor responses during the ‘pre’ and ‘post’ phases. We found that in DAT^ChR2^ mice, the accuracy of the classifier improved after pDA pairing (Fig. 2b). No change occurred instead in DAT^YFP^ mice (Fig. 2b). In line, the plasticity elicited by pDA pairing altered selectively the evolution of the population trajectory of the paired odor response (Fig. 2c). Such deflection had the effect of both increasing the odor response, by increasing its deviation from the resting condition (Fig. 2c) and setting it apart from the representation of other odors (Fig. 2d). We asked, then, whether this enhanced discriminability also increased perceived stimulus salience. We used the sniffing frequency as proxy for the perceived salience of the stimuli^31,32^ and found that sniff frequency increased selectively for the paired odor after evoked pDA release in DAT^ChR2^ mice (Fig. 2e, Supplementary Fig. 5). In summary, pDA was sufficient to induce plasticity to make natural odor stimuli more salient over others, both in their neuronal representations and perception.

### Value assignment to striatal encoding during learning

**Fig. 3:**
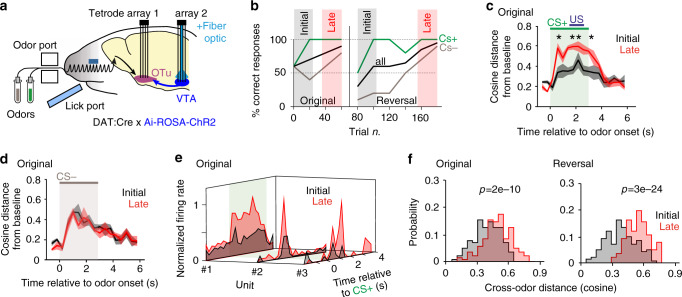
Value assignment to sensory stimuli in the ventral striatum during reversal learning. **a** To assess how stimulus-triggered neuronal responses are modified during reversal learning, we performed recordings in head-fixed mice of VTA and OTu. ChR2-expression in DAN allowed for optogenetic tagging. **b** Exemplary performance of a mouse learning the go-/no-go task (original phase). Once criterion was reached, the odor-reward contingency was reversed (reversal phase) within the session. To reveal changes after learning, each phase was divided into an ‘initial’ phase (comprising the first 12 CS+ and 12 CS− trials) and a ‘late’ phase (last 12 CS+ and 12 CS− trials). **c**, **d** The cosine distance of the population vector from baseline changed for CS+ (but not CS−) from the initial to the late trials (two-sided *t*-test, asterisks mark significance at *α* = 0.05 with Benjamini–Hochberg correction). Displayed mean ± S.E., *n* = 4 trial-averages of three trials, respectively, for both ‘initial’ and ‘late’. **e** Examples of normalized PSTH of responses to CS+ in the initial and late trials in 3 SPN. **f** Distribution of cosine distances between CS+ and CS− representation in initial and late trials, respectively. During learning, CS+ and CS− representations diverged both in the original and reversal phase (two-sided *t*-test). Source data are provided as a Source data file. See also Supplementary Figs. 6-7.

In the next step, we tested whether the plasticity observed in SPN by evoked pDA release matched changes in stimulus representations in these neurons during stimulus-reward learning, and whether the plasticity in the stimulus responses in SPN may generate a reward prediction signal driving DAN during association learning. We examined this question in an odor-guided reversal learning go/no-go task and recorded simultaneously from the OTu and VTA (Fig. 3a, Supplementary Fig. 6a). During recordings, mice learned to lick during the rewarded odor and not to lick during the non-rewarded one. Once the animal reached criterion (80% correct responses over 50 trials), reward contingencies were reversed within the same session and the new association was learned (Fig. 3b, Supplementary Fig. 6b).

To assess plasticity in CS representations before the onset of reward, odors were presented for 3 s and the reward could only be released by licking three times 1.5 s after odor onset. Under this specific condition, 28 out of 75 recorded units had features of putative SPN and many responded to CS (Supplementary Fig. 7a-c). To study the effect of learning on odor encoding, we compared the neuronal responses of SPN in the first 12 trials of the session when animals were unsure about the association of odor and reward, with the last 12 trials when animals had reached criterion (Fig. 3b). The SPN population vector analysis revealed that the neuronal representation to CS+ became more distinct from baseline when animals learned to assign value to the stimulus, while no changes occurred in response to CS− (Fig. 3c, d, Supplementary Fig. 7d). At the single-unit level, excitatory responses to CS+ increased (Fig. 3e). Moreover, as predicted, the population vectors of the responses to the rewarded odor and those to the non-rewarded odor became more dissimilar after learning (Fig. 3f, Supplementary Fig. 7e,f). During the reversal phase, a similar effect was observed (Fig. 3f, Supplementary Fig. 7g). Re-learning of odor-reward associations increased the odor response to the now rewarded odor, while slightly decreasing the response to the non-rewarded one (Supplementary Fig. 7h,i).

### SPN lead DAN in interregional assemblies

**Fig. 4:**
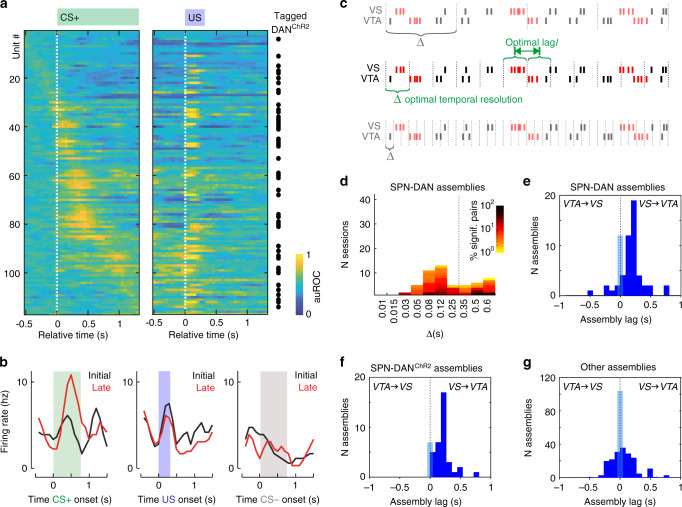
SPN lead DAN activation. **a** Response traces of putative DAN computed with a sliding-window auROC (response vs. baseline). The distribution of optogenetically tagged neurons supports the classification of DAN. **b**, Mean activity for the ‘initial’ 12 vs. ‘late’ 12 trials of the original phase of an exemplary DAN. The plots show an increase in firing at CS+ and decrease at US with learning (moving average smoothing over three adjacent bins). **c** Scheme illustrating assembly detection of interregional assembly (marked in red) with sequential activation. The lag (*l*) measures the delay between the activation of the units composing the assembly. The temporal resolution (Δ) captures the temporal precision/duration of unit activity when firing within the assembly. **d** Distribution of the temporal resolutions of the 359 detected SPN–DAN assemblies. The percentage of significant pairs of all possible pairs for each session is color-coded. SPN–DAN pairs had two characteristic time scales. **e**–**g** Distribution of inter-unit activation lags of assemblies with Δ < 250 ms. Positive lags indicate that the VTA unit followed the activation of the VS unit (and vice versa). Directionality was observed for (**e**) the 63 SPN–DAN and (**f**) 43 SPN–DAN^ChR2^ assemblies, in contrast to (**g**) 296 assemblies composed of other cell types. Source data are provided as a Source data file. See also Supplementary Figs. 8.

The plasticity observed during association learning matched the pDA-induced plasticity. During stimulus–outcome learning, SPN responses to CS+ increased and moved away from the responses to CS−. We therefore tested whether this enhanced network response to CS+ may generate a reward prediction signal conveyed from SPN to DAN. To this aim, we recorded 878 additional units from 68 different sessions. To make sure that the obtained results did not depend on a specific task setting, sessions were performed with longer and shorter stimulus presentations (1–3 s) and delays of the retrieval window (0.75–1.5 s) (see Methods). DAN were classified according to previously established criteria^5^ on the basis of their transient reward and stimulus responses (see Methods) and confirmed by optogenetics-assisted tagging in these animals (Fig. 4a, b, Supplementary Fig. 8).

To study the formation of functional assemblies in the VS–VTA circuit, we used a cell assembly detection algorithm (CAD*opti*, see Supplementary Information) that enables the identification of arbitrary spike activity patterns that repeat above chance level (hereafter termed ‘assembly activation’). Both the time lags *l* between the activation of assembly units and the precision Δ of their coordination are free parameters estimated from the experimental data (Fig. 4c). Importantly, the method automatically corrects for non-stationarities at multiple time scales typical of learning paradigms.

The analysis of assembly-pairs revealed that SPN and DAN formed functional assemblies at two main time scales: a sharper one that peaked around 100 ms and a broader one, with firing rate coordination at or above 600 ms (Fig. 4d). We further analyzed only assemblies with temporal precision smaller than 250 ms as compatible with reward prediction signaling. The distribution of the time lags between the activation of the two assembly units peaked at a lag of 200 ms (Fig. 4e). The lag was directional with SPN leading DAN. This directionality was confirmed and even more pronounced when testing only assemblies with optogenetically tagged DAN (Fig. 4f). Interestingly, assemblies composed of other putative striatal and VTA cell types than SPN and DAN, did not display a preferential directionality (Fig. 4g). Thus, during the learning paradigm directional SPN–DAN assemblies are in a position to transfer information from VS to VTA.

### SPN–DAN assemblies emerge with stimulus–outcome learning

The hypothesis that SPN–DAN directional assemblies encode reward prediction signals comes with two predictions: First, SPN–DAN assemblies activate preferentially to CS+ compared with CS−; second, SPN–DAN assembly activation emerges during learning of the stimulus–outcome association.

**Fig. 5:**
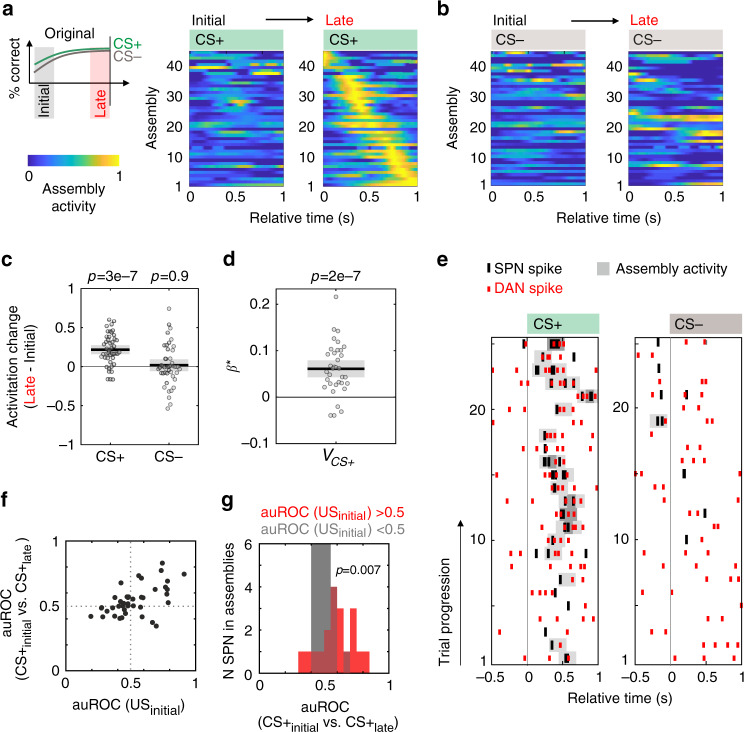
SPN–DAN assemblies emerge with learning. **a**, **b** Activity of directional SPN–DAN assemblies after CS+ and CS− onset. To capture learning, activity was separately averaged for the 12 initial and 12 late trials (original phase). Same normalization for each assembly across the four panels. **c** Difference in mean activity between late and initial trials of directional assemblies at CS (original phase, two-sided Wilcoxon test, *n* = 45, data displayed as mean ± S.E.). Throughout this figure: CS window = 0–0.7 s from CS onset. **d** Standardized and transformed β* coefficients of the Poisson regression of assembly activity at CS+ on the subjective value *V*_CS+_(*t*) assigned by the animal to CS+ at each trial of the original and reversal phase. *V*_CS+_ was estimated from the behavioral data with a Q-PH*f* reinforcement learning model (see Methods). β* coefficients were greater than zero (two-sided *t*-test), confirming a positive correlation between SPN–DAN assembly activity and value assignment. Regression performed on all SPN–DAN assemblies. Among the 31 assemblies with positive β*, 26 had lag>0. Only significant β* displayed (*n* = 35, data displayed as mean ± S.E.). **e** Peri-stimulus raster plot of a SPN and a DAN forming an assembly correlated with *V*_CS+_ (assembly activity shown in gray, darker = stronger activation). Assembly with Δ = 0.12 s and lag $l = 1{\mathrm{\Delta}}$ (SPN preceding DAN). **f** For SPN participating in SPN–DAN assemblies, the plasticity at CS+ (${\mathrm{auROC}}({\mathrm{CS }}+ _{{\mathrm{initial}}}{\mathrm{vs}}.\,{\mathrm{CS}} + _{{\mathrm{late}}})$) was compared with the initial activity of SPN at US (${\mathrm{auROC}}({\mathrm{US}}_{{\mathrm{initial}}})$). Each data point marks the mean auROC of spike counts in the original and reversal phase of one SPN. **g** Distributions of ${\mathrm{auROC}}({\mathrm{CS }}+ _{{\mathrm{initial}}}{\mathrm{vs}}.\,{\mathrm{CS}} + _{{\mathrm{late}}})$ for SPN with excitatory (${\mathrm{auROC}}({\mathrm{US}}_{{\mathrm{initial}}})$>0.5) or inhibitory (${\mathrm{auROC}}({\mathrm{US}}_{{\mathrm{initial}}})$<0.5) response to US. SPN with an excitatory response to US in the initial trials showed a reinforced response to CS+ in the late trials (two-sided Wilcoxon test). Source data are provided as a Source data file. See also Supplementary Figs. 9–11.

To test for these predictions, we analyzed the assembly activation during the task. Most SPN–DAN assemblies displayed significantly more stimulus-related activation during CS+ triggered hit trials than CS− triggered correct rejection trials (Supplementary Fig. 9a,b). To capture the evolution of such activation, we compared the assembly activity during the initial 12 and the last 12 trials of the learning of specific stimulus–outcome pairs (Fig. 5a). While no change was observed for CS− trials, the activity of SPN–DAN assemblies significantly increased for CS+ trials in both the original and reversal phase (Fig. 5a–c, Supplementary Fig. 9c-f). In addition, in the reversal phase, responses to the now non-rewarded odor tended to decrease (Supplementary Fig. 9c,d). Thus, the selective activation to CS+ compared to CS− evolved only when animals learned the stimulus–outcome association (Supplementary Fig. 9e,f). We also tested whether these assemblies would occur with different durations of the stimulus presentations (1, 1.5, or 3 s) or delays to the onset of the retrieval window (0.75 or 1.5 s). Directional SPN–DAN assemblies occurred robustly with comparable likelihoods in all sessions (Supplementary Fig. 9g-h). In the assembly analyses, we therefore pooled sessions with different odor durations and reward delays.

### SPN–DAN assembly activity represents learned stimulus value

The emerging selective activation to CS+ during learning is in line with SPN–DAN assemblies encoding the subjective value assigned to the stimulus. To further prove this hypothesis, we explicitly tested if the activity of these assemblies correlated with the value *V*_CS+_(*t*) assigned by the animal to CS+ at each trial. Value updates were tracked by a reinforcement learning model fit on the behavioral data of each animal. The model generalized a Q-learning model^33^ with a hybrid Rescorla–Wagner rule and Pearce–Hall update mechanism^34–36^ by adding a time-dependent forgetting component^37^, and was selected among three alternative models for its best fit of the behavioral data (for discussion of the model and model selection see Methods and Supplementary Fig. 10a). The value *V*_CS+_(*t*) assigned to the conditioned stimulus at each trial was regressed on the activity of the assemblies during the first 0.7 s from CS+ onset (see Methods). As predicted, SPN–DAN assembly activity correlated positively with *V*_CS+_ (Fig. 5d). Again, the relative occurrence of assemblies significant for *V*_CS+_ did not depend on the duration of the odor presentation or on the delay time to the reward retrieval window (Supplementary Fig. 10b). Further, as expected, the representation of CS+ changed during learning both in SPN and DAN composing the assemblies significant for *V*_CS+_ (Fig. 5e and Supplementary Fig. 10c,d). While learning increased significantly CS+ responses in SPN participating in directional assemblies, this plasticity was only weakly expressed in the population of SPN with excitatory responses to CS+ that did not participate in directional assemblies (Supplementary Fig. 10e-g). SPN with inhibitory responses to CS+ did not change with learning. Together this may suggest some functional specialization of the assembly-forming SPN within VS. In summary, we observed that during stimulus–outcome learning, the firing of SPN to CS+ is reinforced to inform DAN in interregional assemblies about the predicted reward.

We finally examined whether the interregional activity is compatible with a model in which DA could reinforce the response to CS+ in SPN during initial trials of the association learning. During initial trials of association learning, pDA is prominently elicited at US and predicted to function as a reinforcement signal in OTu of the VS^1,16^. SPN responses to stimuli were relatively long and lasted often until US (cf. Fig. 3). Indeed, SPN in assemblies with excitatory responses at CS+ showed also preferentially excitatory responses at US in the initial trials (Supplementary Fig. 11). It is, therefore, possible that pDA release at US could serve as initial reinforcement signal to enhance responses to CS+ in SPN and thereby initiates the formation of the reward prediction signal. Consistent with this scenario, SPN with excitatory responses at US in initial trials also displayed the most pronounced plasticity at CS+ (Fig. 5f, g). These observations support the concept that the initial reinforcement of CS+ in VS are at least in part mediated by DA release at US.

## Discussion

The present study investigates the plasticity in SPN–DAN interactions for the encoding of stimulus-triggered reward prediction signals. Behaviorally, the reinforcement of conditioned stimuli depends on phasic DA. As a neuronal correlate, we demonstrate that phasic DA is sufficient to induce plasticity in the SPN population encoding the paired odorant even in the absence of reward. At the level of single-unit responses, pairing with phasic DA increased the intensity of excitatory stimulus responses in SPN, a modification that is compatible with DA-induced synaptic plasticity of SPN in vitro^17–19^. Specifically, in a recent study in acute slice preparations of the adult OTu, SPN displayed postsynaptic potentiation upon pairing with phasic DAN stimulation and consequently an increased firing output to olfactory input stimulation^19^. The plasticity induced by optogenetic DAN stimulation was blocked by D1 receptor type antagonists. Further, pharmacologic D1 receptor activation was sufficient to induce plasticity. Thus, DA is necessary and sufficient to induce plasticity in olfactory input synapses of SPN. Besides dopamine, DAN also co-release the fast neurotransmitter glutamate to OTu and thereby modify firing of striatal cholinergic interneurons^38^. Future studies may clarify whether such fast co-release from DAN may potentially further promote SPN plasticity.

At the population level, the plasticity pushed the network representation of the paired stimulus to stand out both from the resting state activity and from other odor representations. Such modifications were selective to the odor paired with phasic DA; hence, leaving the responses to other non-paired odors unchanged. Compatible with the concept that VS encodes the value of stimuli^13,24,39–41^, the decoding of stimulus representations increased after pairing with phasic DA. This increased distinctness of stimulus representation was also reflected in the perceived salience of the stimulus paired with phasic DA. In line with selective stimulus reinforcement, only the paired stimulus displayed a sustained increased sniffing response indicating a heightened perceived salience. Together, these findings provide a neuronal plasticity correlate of the previous behavioral observations that phasic DA release to both the nucleus accumbens or the olfactory tubercle of the VS transforms neutral stimuli into conditioned ones and elicits behavioral preference^20,22^. The present findings also reveal a central function of the olfactory tubercle among the specialized circuits of the ventral striatum in transforming external world stimuli into reward prediction signals.

Remarkably, while the observed plasticity in VS was exclusively the result of the stimulation of DAN terminals, a similar selective reinforcement of SPN stimulus representations was also observed for the rewarded stimulus during stimulus–outcome learning. This latter finding matches other recent observations that pairing with reward enhanced odor responses in SPN of the OTu^42^ and their immediate early gene expression^43^; taken together these results highlight the role of the OTu as a region in the VS where sensory input is transformed into expected value signals to guide behavior. Phasic DA-release occurs at unexpected rewards as a positive salience signal^1,44^. Thus, during the initial trials of stimulus–outcome learning, SPN representations of CS+ could be reinforced by DA bursts released at US. Indeed, SPN active at US in the initial trials were also those with higher plasticity in their CS+ responses. It is certainly possible that additional phasic DA signals, for instance those evoked by stimulus novelty, may contribute as well to VS plasticity. Future studies designed to disentangle the respective contributions of DA signals at CS and US may detail this question.

Based on the dopaminergic reinforcement of stimulus representations in VS, we investigated whether these enhanced representations of CS+ could drive VTA firing for the encoding of reward predictive signals. VS has been proposed to influence dopaminergic discharge in reinforcement learning^35,45^. VS sends outputs to VTA^10,46,47^ that will subsequently activate DAN and elicit stimulus-locked conditioned behavior^25^. To directly examine physiological interregional VS–VTA interactions in dynamic reinforcement learning, we performed simultaneous dual-site recordings from VS and VTA. We had therefore designed a dual-site recording array adapted to the anatomical geometry. Interregional assemblies between SPN and DAN were then identified through CAD*opti*, a further development of the unsupervised mining technique CAD for the detection of assembly activations^48^. CAD*opti* could reveal even sparse spiking patterns as in SPN–DAN assemblies, owing to the implemented non-stationarity correction. Characteristics of SPN–DAN assemblies were estimated from the data. These characteristics fulfilled criteria to communicate reward predicting stimulus signals from VS to VTA. First, the temporal precision and the delay in activation after stimulus onset matched the valuation component of reward prediction signals^1,9^. Second, the assemblies were directional with SPN activation leading DAN firing. Within the assembly activation, SPN and DAN had a lag of about 100–200 ms. As SPN are inhibitory neurons, DAN activation by SPN firing is thought to be mediated predominantly through disinhibition via intermingled GABAergic neurons in VTA or ventral pallidum^46,47,49^. In principle, direct inhibitory inputs can also elicit rebound bursting with a delay in DAN. Yet, subsets of SPN in nucleus accumbens that project directly onto DAN^10,49^, inhibited DAN firing, but did not elicit bursts^49^. The subset of SPN found in nucleus accumbens directly inhibiting DAN suppress behavior, while the SPN evoking DAN firing through disinhibition promote reward-related behaviors^49^. In the OTu, SPN in directional assemblies drove firing in DAN with a lag compatible with di-synaptic disinhibition or, less likely, transient monosynaptic inhibition followed by rebound bursting.

In line with directional SPN–DAN assemblies conveying the information of predicted reward from CS+, assembly activity developed during stimulus–outcome learning. We captured the dynamics of learning with a reinforcement learning model fit to the animals’ behavioral choices. SPN–DAN assembly activation correlated positively with the evolution of the value associated by the animal to CS+. Timing, amount and probability of reward are known to all add to the subjective value of a stimulus^26,50^. Future studies may dissect further whether SPN–DAN assemblies encode multiple or single factors contributing to the subjective value.

In summary, our data provide evidence for a model favoring a central role of VS in reinforcement learning, whose bidirectional communication with VTA computes the prediction component of the RPE. As a novel stimulus is paired with an unexpected reward, DAN fire bursts at US. As a consequence, pDA is released in the VS, reinforcing those SPN that responded to sensory stimuli and are still active during US. As learning progresses, the strengthened SPN response to CS+ gradually drives the firing of DAN to CS+, to encode the reward prediction component of the RPE. These SPN-recruited DAN, then, release DA back to VS that is suited to maintain reward prediction responses as long as the stimulus–outcome association exists. These reinforcement processes in the VS–VTA loop for predictive value assignment may be crucial to functional and dysfunctional social behaviors^51,52^ and for the development of mental disorders as drug addiction^53^ and psychoses^54^.

## Methods

### Animals and husbandry

DAT:(IRES)Cre mice (B6.SJL-*Slc6a3*^*tm1.1(cre)Bkmn*^/J; RRID: IMSR_JAX:006660)^55^ were maintained in a heterozygous C57BL/6J (Charles-River) background. For the optogenetic tagging in mice performing the reversal learning task, we crossed DAT:(IRES)Cre^+^ with Ai32(RCL-ChR2(H134R)/EYFP) mice (B6;129S-*Gt(ROSA)26Sor*^tm32(CAG-COP4*H134R/EYFP)Hze^/J)^56^ to obtain heterozygous mice for both alleles expressing ChR2-YFP in DAN. Expression of ChR2:YFP fluorescence was restricted to DAN (not shown). Mice were single housed upon implantation of the recording array at a 12-h day-and-night cycle (room temperature ~24 °C, air humidity 45–50%). Recordings were performed with male mice that were 3–6 months old. All procedures were approved by the Regierungspräsidium Karlsruhe, State of Baden-Wuerttemberg, Germany and in accordance with NIH guidelines.

### Virus preparation

To generate cell-type specific expression of ChR2, we injected a Cre-inducible recombinant adeno-associated virus (AAV) vector rAAV_1/2_-double floxed(DIOA)-EF1a-hChR2(H134R):mCherry-WPRE-HGHpA (RRID:Addgene_20297)^57^ into heterozygous DAT:Cre mice. For control experiments, DAT:Cre mice were injected with a rAAV_1/2_-DIOA-EF1a-eYFP virus (RRID:Addgene_27056). Cre-inducible recombinant AAV vectors were produced in-house using CRL-11268 293T/17 HEK cells (ATCC LOT:62312975) with AAV1/2 coat proteins (plasmids gift of M. Klugmann) to a final viral concentration of 10^16^ genome copies/ml after purification over heparin columns (GE HiTrap Heparin HP columns). DAT:Cre positive mice 8 weeks of age were anesthetized with isoflurane; and 0.75 µl of purified virus was injected into each hemisphere of the ventral midbrain (location from Bregma: posterior, 3.0 mm; lateral, 0.8 mm; ventral, 4.4 mm). All mice recovered for at least 21 days before undergoing implantation of the recording array. Expression was confirmed in post hoc histological exam. Selectivity of viral overexpression to TH positive ventral midbrain neurons had been previously determined^19^ and was confirmed in the present cohort (not shown), generating mice with ChR2:mCherry expression in dopaminergic neurons (DAT^ChR2^ mice) or control mice (DAT^YFP^).

### Recording array

For the recordings during reversal learning, the OTu and VTA were targeted unilaterally with 16–20 tetrodes per array and for the DA plasticity experiment each array contained up to 32 tetrodes. Given the depth of the target structures (>5 mm) and curvature of the OTu (Supplementary Fig. 1b), we developed an array to address these challenges with a flexible design adaptable for single- or multi-site configurations (Supplementary Fig. 1c-f). The design exploits the high precision of printed circuit boards (PCB) (±10 µm, Würth Electronics) to achieve accurate x–y placement of the guiding channels for tetrodes (spun from 12.5 µm teflon-coated tungsten wire, California Fine Wire) and optical fibers (Thorlabs FT-200-EMT). As a building scaffold, six identical PCBs were stacked precisely on top of each other via 26-gauge steel tubing threaded through four holes placed peripherally. This allowed for an easy placement and parallel alignment of multiple recording electrodes (and optical fibers) in the guiding channels formed by the aligned holes. An identical PCB was stacked on top of the scaffold and equipped with a soldered-on Molex SlimStack connector (mated height: 1 mm, pitch: 0.4 mm, 70 channels, model n. 502426-7030), providing the implanted electrode interface board (EIB). For our application, the board was placed above a molding of the rostro-ventral brain surface, so tetrodes and an optical fiber matched the bowl-shaped 3D-curvature of the OTu. The tetrodes were then fixed in place with a drop of acrylic adhesive. The single wires were connected by threading them through 200 µm through-hole vias to achieve reliable mechanical and electrical contacts with the EIB. To protect them from damage, they were encased in two-component epoxy. After assembly of the recording arrays, tetrode tips were gold-plated with a NanoZ-device (Multichannelsystems) to target an electrode impedance of 300 kOhm at 1 kHz. To connect to the Intan RHD2164 head stage during recordings, a custom-built adapter from the Molex SlimStack connector to two 36 Omnetics Nano Strip connectors was used.

### Implantation of recording array

Mice were anesthetized with isoflurane and pre- and post-surgery analgesia was administered. A roughly circular patch of skin above the skull was removed. Local anesthesia was applied to the skull. The lateral and nuchal muscle insertions were left intact. The operative field was then prepared by attaching the margins of the skin to the circumference of the top of the skull with VetBond (3 M), to protect soft tissue from damage, contamination or necrosis, and leaving only the surface of the skull exposed. Holes were drilled in the skull above the regions where tetrodes and fiber optics were inserted and for grounding above cerebellum. The skull was then coated with Super-Bond C&B (Sun Medical) following insertion of a small Neuralynx gold pin as ground connected to the recording array through an insulated copper wire. The tetrode array was slowly lowered into the brain with a motorized 3-axes micromanipulator (Luigs & Neumann). The center of the tetrode bundle was targeted from Bregma for the OTu: anterior 1.6 mm, lateral 1.3 mm, and ventral 4.9 mm from dorsal brain surface, and for VTA: posterior, 3.1 mm lateral, 0.5 mm, and ventral, 4.4 mm from dorsal brain surface. After reaching target depth, dental cement (Kulzer Palladur) was applied at the margins of the recording array so that gravity and capillary force ensured complete filling of the narrow gap between the bottom of the array and the adhesive-coated skull. After the cement hardened, animals recovered in their home cage. The entire surgical procedure took ~1 h. Animals were normally fit and eating after <30 min.

After completion of the recordings, animals were euthanized and perfused with 4% PFA and postfixed for at least 2 weeks in toto comprising the brain, skull, and implant. Sectioning confirmed rostrocaudal and mediolateral placement in the borders of the OT and VTA (Supplementary Figs. 1a,b and 6a). Due to low levels of scar formation and microglia activation with the tetrode array, we could not reliably detect tetrode tracts in histological sections with Nissl stain or immunohistochemistry against glial proteins (not shown).

### Recording configurations

After recovery, the mice were placed in the head-fixed setup. The first few sessions were brief (5–20 min) and served to habituate the animals to head fixation in the setup. Mice accommodated typically after 2–3 sessions to head fixation. Then, odor sessions started. For odor delivery, a custom-built air-dilution olfactometer was used (Shusterman et al.^58^). Odors were kept in liquid phase (diluted 1:100 in mineral oil) in dark vials and mixed into the nitrogen stream that was further diluted 1:10 into a constant air stream in the olfactometer. The following natural flower odors were used in the dopamine plasticity experiments: geranium and ylang-ylang (Sigma-Aldrich W250813 and W311936, respect.). The two odors at the chosen concentration evoked in recordings from the OTu comparable responses. Odors were delivered in a pseudo-randomised order. Odors were applied for 500 ms with an inter-trial interval of 10 s. Recordings were performed with two Intan 64 channel RHD2164 miniature amplifier boards connected to a RHD2000 interface board and open-source Intan interface software. Inputs from the laser, olfactometer and sniff sensor were simultaneously recorded with the same interface board, as were reward application and licking activity (via an infra-red beam break sensor positioned in front of the licking spout, Omron Electronics EE-SX3009-P1). Data were sampled at 30 kHz.

### Sniff recording

To monitor the sniff signal, we used a custom-built snout mask that was gently pressed against the snout to generate a cavity in the mask in which pressure fluctuations were continuously measured through a HDI pressure sensor HDIM020GBY8H3 from First Sensor Inc. connected to the analog input of the Intan RHD2000 interface board. The mask was modified based on the original design of Dmitry Rinberg. The influx of odorized air into the cavity of the mask was calibrated to the outflow through continuously measured vacuum. The mask allowed for measurement of the breathing cycle as the system was calibrated to keep the (odorized) air inflow constant relative to the outflow. The animals quickly adapted and tolerated the pressure mask.

The sniff signal was converted and analyzed with custom-written MATLAB scripts using a 1–50 Hz band pass filter. For monitoring changes in the sniff frequency, we analyzed the duration of two full sniff cycles before and after odor onset. Averages were formed for each recording the ‘pre’ and ‘post’ phase of each recording session. Throughout the experiment, the baseline sniff frequency remained constant (Supplementary Fig. 5a,b). An initial small increase in response to the odor presentation habituated over the duration of the recording session in DAT^YFP^ mice (Fig. 2e).

For sniff aligned data, trials were aligned to the first onset of inhalation after odor presentation. The results in SPN for DA-induced plasticity were also observed after sniff alignment (Supplementary Fig. 2f).

### Optogenetic plasticity experiments

Awake head-fixed mice were habituated to the recording setup for at least a week to minimize distress and movement artifacts. To obtain efficient DA release, we used viral overexpression of ChR2 in DAN that we had found to produce sharp DA-release transient in OTu upon stimulation with brief laser bursts^38^. The plasticity experiments involving evoked phasic DA release followed a design with three phases: Two odors were applied in a pseudorandomized fashion for 500 ms with an inter-trial interval of 10 s. In each phase no odor was consecutively applied more than three times in a row. We initially pseudo-randomly varied the number of trials per phase around 30. In later experiments we consistently used 40 trials in the first phase (the first 10 were omitted for the analysis), 30 trials in the second phase and 30 trials in the third phase. A further extension of the third phase was not possible in our experimental conditions as in sessions longer than 50 min in total, mice became frequently drowsy and neuronal baseline firing rates changed globally (data not shown). The odor responses before pairing with evoked DA release were recorded in the ‘pre’ phase, followed by a ‘pairing’ phase during which one of the two odors was paired with a train of 5 ms laser pulses at 40 Hz for 300 ms starting simultaneously with onset of the odor application. TTL-driven laser pulses (5 ms duration, 2–5 mW at fiber tip) were delivered from 200 µm multimode optical fibers (Thorlabs) coupled to a 25 mW, 473 nm, diode-pumped solid-state laser. One of the two odors was paired with pDA per session. In the same animal, we repeated the experiment after a week with pairing to the other odor. Data were then pooled for further analyses. To then monitor changes in responses, odors were continued to be applied in the ‘post’ phase. Usually two sessions were performed in each DAT^ChR2^ or control DAT^YFP^ mouse, and the odor that was paired with the ‘pairing’ laser, was switched from session to session. A second laser provided ‘sham’ stimulation above the head of the animal with the same intensity and frequency in all other trials, except when the ‘pairing’ laser was on. In addition, blue light illumination was constantly provided in the recording chamber.

### Behavioral conditioning in the go/no-go task

We trained the animals in the head-fixed setup described above. Mice received water in their home cage so that their body weight stabilized at 85% of baseline body weight. The training comprised multiple stages and progressed after reaching a performance criterion defined as at least 80% correct responses in 50 consecutive trials. Trials were considered correct if either at a ‘go’-response the reward was retrieved, by licking at least three times within a fixed window of time (‘retrieval window’), or at a ‘no-go’-response no licking was detected during the retrieval window. In the initial sessions, the animals’ licking behavior was shaped by first presenting them with a drop of water and subsequently letting them obtain more water when they licked at the licking spout (available in a random interval schedule, 0.5–12 s). Stage 1: A single odor (1.5 s stimulus duration) was presented. Animals could obtain a 5-µl drop of water if they licked at least three times during a window from 0 to 2.5 s after odor onset (retrieval window), this was considered a ‘go’-response. The interval between trials was randomly set at 10 ± 2 s in all stages. Stage 2 (‘discrimination’) consisted of two odors in pseudo-random succession (no more than three consecutive trials with the same stimulus). One odor (1.5 s duration) was rewarded as in stage 1 (retrieval window: 0.5–2.5 s), while a ‘go’-response for the second odor was registered as a false alarm. No punishment was used. Stage 3 (‘reversal learning’) used the same parameters as stage 2, but upon reaching the performance criterion (in the ‘original phase’), the reward contingency of the odors was switched (‘reversal phase’). The dataset used in this study comprises recordings from completed sessions (original and reversal phase) for different paradigm settings. We varied the settings between sessions for the onset of the retrieval window (‘Rw’: 0.75 and 1.5 s) and in the length of stimulus presentation (‘Sp’: 1, 1.5, and 3 s). Only sessions in which the animal reached criterion in the original phase were included in the analysis. Seventy-eight sessions in 11 animals were included in the assembly analysis comprising 1012 single units, of which 413 were classified as SPN and 116 as DAN. Forty-three sessions were performed with Rw = 0.75 s, Sp = 1.5 s; 25 sessions with Rw = 1.5 s, Sp = 1 s; and 10 sessions with Rw = 1.5 s, Sp = 3 s.

### Data pre-processing: spike detection

To reduce noise and movement artifacts affecting all recording sites, we subtracted the median voltage trace of all channels from each recorded trace. The resulting signal was band pass filtered between 300 and 5000 Hz (4th order Butterworth filter, built-in MATLAB function). A threshold value for spikes was computed as a multiple (7.5×) of the median absolute deviation of the filtered signal^59^. Temporally proximal detected peaks over threshold were pruned by height to a minimum distance of 1 ms to avoid multiple detections of the same multiphasic spike. When an event was detected on multiple channels of a tetrode, the timestamp of the highest detected peak was used. Spike waveforms were extracted around −10 to +21 sampling points around the peak.

### Data pre-processing: spike sorting

Spike sorting was done with a custom-built graphical user interface in MATLAB, originally developed by A. Koulakov (CSHL). Metrics used for clustering included detected peak height or amplitude (and the respective principal components over channels), and the first three principal components of the waveforms for each respective channel in case spikes were predominantly recorded on one channel. The quality of single-unit clusters was assessed using the mlib toolbox by Maik Stüttgen (Vs. 6, https://de.mathworks.com/matlabcentral/fileexchange/37339-mlib-toolbox-for-analyzing-spike-data) with particular attention to peak height distribution (fraction of lost spikes due to detection threshold), contamination (fraction of spikes during the refractory period <5 ms) and waveform variance.

### Selection criteria for putative SPN

SPN are strongly modulated in their activity patterns by the state of the animal^60^. To account for the state dependent activity of SPN and facilitate comparison with existing literature, we used separate classification criteria previously established for recordings of SPN in passive paradigms^46,61^ and from active animals performing a task^62,63^. In the awake, passive experiments with evoked pDA, units with <5 Hz baseline firing rate were classified as SPN (Supplementary Fig. 2b) based on features of optogenetically identified SPN^46^. To perform the plasticity experiments, SPN had to comply with a set of criteria: Throughout the analyzed part of the recording session units were allowed to only have a maximum change in baseline firing rate from beginning to the end of the session of <10% and intermittent maximum fluctuations of 20%. After exclusion of the first ten trials of odor application where we frequently observed an initial response habituation, we selected units with stable odor responses for the two odors throughout the ‘pre’ phase. Few units that were responding with short latency to laser pulses in DAT^ChR2^ mice and had features previously described for striatal cholinergic interneurons^63^ were excluded from the analyses (not shown) in agreement with previous molecular tagging experiments^64^. Due to the low expression of ChR2 in the transgenic mice used for the reversal learning, this latter exclusion of cholinergic interneurons was not possible. For this reason and to account for the different activity states of SPN in mice engaged in task performance, we classified VS units according to baseline firing features according to criteria previously used for striatal units recorded during behavior^63^. All units with a firing rate <2 Hz were classified as SPN. All units with a firing rate >12 Hz were excluded as putative fast spiking neurons from the OTu or from the directly neighboring ventral pallidum. Units in the ambiguous range from 2 to 12 Hz were designated as putative regular-firing cholinergic interneurons if the coefficient of variation of their inter-spike interval (ISI) distribution was <1.2 and ISIs of <60 ms contributed no more than 20% of all ISIs^63,65^. The remaining units were assigned as SPN if they ever paused firing for more than 2 s (with a fraction of ISIs bigger than 2 s > 1/10^−4^)^63^. Neurons that did not fulfill this latter criterion were considered fast spiking neurons. The fraction of cholinergic interneurons was in the expected range for ventral striato-pallidal regions (~3%). With this classification, units recorded during reinforcement learning had a baseline firing rate (median ± interquartile range) of SPN: 0.81 ± 1.65 Hz, fast spiking neurons: 35.41 ± 34.67 Hz, cholinergic interneurons: 3.21 ± 1.82 Hz. For the population analysis in Fig. 3, both classification approaches for passive and active states identified the same set of putative SPN.

### Optogenetic identification of DAN

To maximize the yield in optogenetic tagging for cell-type identification, we used heterozygous transgenic mouse lines to express ChR2 in all DAN. This approach allowed for efficient tagging, but was insufficient to trigger efficient DA release (not shown). We identified optogenetically modulated units by cross-correlating spike trains and laser pulses, and comparing the results with a distribution of constructed control cross-correlograms. After each session, trains of 5 ms laser pulses (8–12 Hz, 5 mW) were delivered via implanted optical fibers. For each recorded single unit, a test cross-correlogram was computed with the timestamps of spikes and laser pulses (bin width: 1 ms, lag: 0–20 ms). To test for significant modulation, control cross-correlograms (*n* = 10,000) were constructed by randomly jittering each laser pulse in the interval ±30 ms around its original time, thus eliminating temporal relation on that time scale while preserving the properties of the spike train. Modulation was considered significant if two consecutive bins of the test cross-correlogram were above 95% of the global distribution of the combined control cross-correlograms.

### Classification of VTA units

Single units in the VTA were classified into three types via their task-related activity using a clustering approach adapted from Cohen et al.^5^. Type 1 corresponds in this classification to DAN. First, responses were characterized for the relevant time spans (from −0.5 to 1 s around CS+ and US) in all applicable experimental paradigms: We constructed spike count distributions (over trials) for 200 ms bins shifted by a 50 ms increment in the CS+ and US windows. The control distributions were computed by tiling the 2 s before trial onset with 200 ms bins and merging the resulting distributions. Significant activation or inhibition was assessed with the Friedman test. Only units with a significant response (*p* < 0.05) were included in the clustering for classification. For the clustering of US-responsive units, the spike count distributions were used to construct one trace per neuron by computing auROC-values analogously to the aforementioned significance test, yielding a trace of values between 0 and 1, and with 0.5 meaning no distinguishability between test and control distribution (i.e., response and baseline activity). Hierarchical clustering was done on the basis of these traces, using the built-in linkage function of Matlab (cityblock distance metric using average distance). The activation pattern of the resulting three clusters (see Fig. 4a and Supplementary Fig. 8) mirrors the one shown by Cohen et al.^5^, suggesting biologically meaningful labels for the clustered single units (type I: DAN, type II: GABAergic neurons, type III: glutamatergic neurons). The optogenetic tagging of DAT+ neurons supported the classification of cell types: 46 of 50 tagged neurons included in the clustering were type I and roughly 40% of the 116 type I neurons were tagged (see black dots in Fig. 4a).

### Analysis of single-unit activity

To analyze whether odor response plasticity can also be detected at the single-unit level, we divided SPN in three groups: neurons with an increase, decrease, or no change in firing rate during the 1 s following odor onset. Due to the low baseline firing rate, z-scoring or comparable normalizations were not suitable for SPN. We therefore considered neurons to have an excitatory response if their mean firing rate in the 1 s response window was at least of 1 Hz and increased at least by 20% from the baseline firing rate (time window of 1 s before odor onset), either in the ‘pre’ or ‘post’ phase. In reverse, we counted units to have an inhibitory response if their baseline firing rate was at least 0.5 Hz and the mean firing rate during the 1 s response window decreased by at least 20% compared with baseline, either in the ‘pre’ or ‘post’ phase. To compare ‘pre’ to ‘post’ odor responses (mean rate in the 1 s following odor onset) we used a paired two-tailed Wilcoxon signed-rank test (Fig. 1h and S3e-f).

### Population analysis: the population vector

Considering the relatively small cell count per session due to the numerous selection criteria (see main manuscript), we built spike count population vectors by pooling units from different sessions^12,66,67^. This approach is sensitive to small changes in rate, if coherent throughout the population, and sums the contribution of both excitatory and inhibitory responses. Since we were interested in either paired or non-paired odor responses, we first divided the trials of each session in paired and non-paired trials. Within these two groups, we combined the population vectors of different sessions ${\mathbf{v}}_t^s = [c_t^{s,1}, \ldots ,c_t^{s,n}]$, with $c_t^{s,n}$ indicating the spike count of unit *n* at time bin *t* in session *s*, in an across-session population vector **V**_*t*_. The vector **V**_*t*_ was obtained by concatenating ${\mathbf{v}}_t^s$ matching the trials according to their trial order, ${\mathbf{V}}_t = \left[ {v_t^1;v_t^2; \ldots ;v_t^S} \right]$. For example, **V**_1_ was obtained by concatenating the first trial of all sessions in which the paired odor was presented to the animal, **V**_2_ by concatenating the second trial of all sessions with paired odor, etc. Figures 1c–f, 2a, 3c, d, f, and Supplementary Figs. 2d-f, 4a were produced using population vectors built with such progressive-trial alignment. This trial matching criterion aims to reflect in **V**_*t*_ the temporal progression of the experiment; however, since the units composing **V**_*t*_ were not (all) recorded simultaneously, we wondered whether a different order in trial-pairing across sessions could lead to different results in our analyses. To exclude this possibility we repeated the analyses for 300 different realizations of population vectors ${\mathbf{V}}_t^{{\mathrm{rand}}}$ obtained by randomly permuting the trial order in which the session specific population vectors were aligned (${\mathbf{V}}_t^{{\mathrm{rand}}} = [v_i^1;v_j^2; \ldots ;v_l^S]$). Supplementary Figs. 3a-d and 4b,c were produced using the randomized population vectors. For these latter analyses, the fraction of the 300 performed tests that had a *p*-value <0.05 is reported in the respective figure legend. Finally, since some sessions had different numbers of trials per phase, we built the population vectors with the minimum number of trials available among sessions. This was done by omitting the last trials of the ‘pairing’ and ‘post’ phases and the initial trials of the ‘pre’ phase.

### Population analysis: temporal evolution of the odor response

Figures 1c, d, 3c, d and Supplementary Figs. 2d,f, 7d,h-i show the average deviation of the population vector from baseline following odor onset. Trials were binned as indicated below. To reduce trial-by-trial variability in neuronal response, we divided the trials in groups of 3 and computed the average population vector of each trial-cluster. In this way, we reduced the number of samples available but we obtained more stable population responses. For each trial group, we built a baseline population vector by averaging the spike counts of *n*_*bl*_ consecutive baseline bins. We then computed the distance between the baseline vector and the population vector for each bin during the trial. Absolute distance values are affected by the specific sample of recorded units and therefore can only be compared within the same set of units (e.g., DAT^YFP^ sample). In order to account for changes of the population vector both in direction and in rate, we computed distances with the cosine similarity metric, $d_{x,y}^{{\mathrm{cosine}}} = 1 - {\mathbf{x}} \cdot {\mathbf{y}}/\left\|{\mathbf{x}} \right\|\left\| {\mathbf{y}} \right\|$, and the Euclidean metric, $d_{x,y}^{{\mathrm{Euc}}} = \left\| {{\mathbf{x}} - {\mathbf{y}}} \right\|/l$ (the Euclidean distance was normalized by the vector length *l*). Finally, we tested the first *n*_test_ bins after odor onset for differences in deviation from baseline of the odor responses in the ‘pre’ phase and in the ‘post’ phase with a two-tailed *t*-test. The test was corrected for multiple comparisons (on the multiple bins tested) with the Benjamini–Hochberg correction. Parameters: Behavioral paradigm: binsize = 0.4 s; *n*_*bl*_ = 3 (between −1.8 and −0.6 s from odor onset); *n*_test_ = 12. Passive paradigm: binsize = 0.25 s; *n*_*bl*_ = 4 (between −1.75 and −1 s from odor onset); *n*_test_ = 7.

### Population analysis: change in odor encoding after pDA pairing

As for the previous analysis, trials were binned in time and averaged in groups of 3. To investigate the effect of pDA on odor responses, we built an odor response population vector by averaging spike counts across the response bins (from 0.6 to 1.8 s from odor onset in the behavioral paradigm and from 0.5 to 1.25 s in the passive paradigm). We then computed the distance (both cosine and Euclidean) between all population vectors of each trial-cluster of the ‘post’ phase and ‘pre’ phase and between all population vectors of the ‘pre’ phase within each other. In this way, we could test if the distance between the odor responses in the two phases was larger than the original trial-by-trial variability. We finally tested for interaction between cohorts (DAT^ChR2^, DAT^YFP^), odor type (paired, non-paired), and phase (‘pre’,’post’) through a three-way ANOVA. Post hoc comparisons were performed with Tukey’s test correction for multiple comparisons (factors: mice group (levels: DAT^ChR2^, DAT^YFP^), phase (levels: ‘pre’,’post’), and odor (levels: paired, non-paired)) in Fig. 1e, f, Supplementary Figs. 2e and 3a-d.

### Population analysis: change in distance between odor encodings after pDA pairing

To test whether the distance between the neural representations of the two odors increased after DA pairing, we computed the distance between the response population vectors (as described above) to the two odors both during the ‘pre’ phase and ‘post’ phase. Distances were computed with both cosine and Euclidean metrics. Significance was tested with a two-tailed *t*-test in the behavioral paradigm (where no control group was present, Fig. 3f, Supplementary Fig. 7E-G) and with a two-way ANOVA with post hoc Tukey’s test correction in the passive paradigm (factors: mice group (levels: DAT^ChR2^, DAT^YFP^) and phase (levels: ‘pre’,’post’)) in Fig. 2a and Supplementary Fig. 4.

### Population analysis: odor response classifier

We tested if pDA improved odor discriminability by measuring the accuracy of a classifier in discriminating the odor responses of the paired and non-paired odors in the ‘pre’ phase and in the ‘post’ phase (Fig. 1a). Trials were divided into four groups based on the odor (paired vs. non-paired) and of the session phase (‘pre’ vs. ‘post’). Both in the ‘pre’ and ‘post’ phase we performed a quadratic discriminant analysis (QDA) to classify paired and non-paired responses. The first step of QDA is to estimate the parameters of, in this case, two multivariate Gaussians representing the data of the training set. The number of parameters *df*_*c*_ per Gaussian component scales quadratically with the dimension *D* of the vector space (specifically $df_c = D(D - 1)/2 + 2D + 1$, yielding in our case $df_{{\mathrm{total}}} = 2 \ast df_c - 1$). Hence, to avoid overfitting, we first reduced the population vector space dimensionality through principal component analysis performed on the four odor response groups simultaneously (making sure that odor representations in the ‘pre’ and ‘post’ phases shared the same space). The classifier was tested with a leave-one-out cross-validation procedure, with total accuracy given as $a = \# {\mathrm{correct}}/\# {\mathrm{tested}}$. To make sure that the results obtained did not depend on the specific dimensionality of the population space, we repeated this analysis for all dimensions from 3 to 6 (for dim>6, the number of the model free parameters started to exceed the number of data points). Finally, we used Fisher’s exact test to assess whether the number of odor responses correctly assigned by the classifier in the ‘pre’ and ‘post’ phases differed. Significance was corrected for multiple comparisons (Benjamini–Hochberg correction) across the four tested dimensions.

### Population analysis: visualization of population trajectories

Trials were divided according to the paired and non-paired odor, and according to the phase of the session: ‘pre’ and ‘post’. Within each of these four groups we averaged the population activity across all trials obtaining four multivariate time series spanning the trial duration. To better unfold neural dynamics^68,69^, we then applied delay embedding and expanded the state space of the neural trajectories adding, for each unit, *m* delayed coordinates (*m* = 3; delay = 1 bin). Finally, to visualize the trajectories, we applied factor analysis (FA) and reduced the dimensionality of the space to 3 (Fig. 2c). FA allows describing the observed variables in terms of a reduced number of independent latent factors. To improve visualization, the trajectories were rotated within the axes.

### Population analysis: visualization of odor responses

Odor response population vectors were defined as explained in ‘Change in odor encoding after pDA pairing’ and divided in four groups according to odor type and session phase. For visualization purposes we reduce the vector space dimensionality to 3 (Fig. 2d). To better appreciate within and between group variability we used multidimensional scaling (MDS) as dimensionality reduction method. MDS is a non-linear dimensionality reduction method designed to preserve in the reduced space the pairwise distances between points of the original space. To improve visualization, we rotated jointly the position of the points within the axes.

### Assembly detection and pruning

Assemblies were detected through CAD*opti*, an unsupervised statistical machine learning framework for non-stationarity-corrected detection of cell assembly at their optimal time scale. CAD*opti* extends from the cell assembly detection algorithm (CAD)^48^. CAD processes the multivariate neuronal time series at a set of user defined time scales ${\mathbf{D}} = \left[ {{\Delta}_{{\mathrm{min}}},...,{\Delta}_{{\mathrm{max}}}} \right]$ and, thanks to the non-stationarity corrections, detects, at each time scale, only those assemblies whose temporal coordination matches the targeted resolution. Despite such strong filtering, if the range of temporal resolutions to test $\{{\Delta}_{{\mathrm{min}}},...,{\Delta}_{{\mathrm{max}}}\}$ is densely sampled, it is possible that assemblies with internal temporal coordination Δ* are detected, albeit sub-optimally, also for a set of temporal resolutions Δ* ± *ε* neighbouring Δ*. To avoid this redundancy and select the most representative time scale, in CAD*opti* we pruned the detected assemblies and kept, for each unit pair, the time scale correspondent to the lowest *p*-value of detection (algorithm available at https://github.com/DurstewitzLab/CADopti). Since we were interested in the directionality of interregional assemblies we set the algorithm to detect assemblies up to unit pairs. This was done to later classify assemblies according to the type of their composing units (specifically targeting assemblies composed by a SPN and a DAN) and study the distribution of the time lag between the activation of the two cell types. Tested time scales: $\Delta \in \left\{ {0.01,0.015,0.03,0.5,0.8,0.12,0.25,0.35,0.5,0.6} \right\}{\mathrm{s}}$; maximal lag tested per time scale, respectively: $l \in \{ 20,20,20,20,20,10,7,5,5,5\}$ bins. With the chosen Δ, the smallest detectable lag is 10 ms (=1 bin of highest temporal precision). Lags <10 ms would fall into *l* = 0. Reference lag^48^ used: $l^ \ast = - 2$.

Assembly detection was performed on the whole recorded time series, so that assemblies could be detected irrespective of their time of activation in the task. The distribution of SPN–DAN assembly resolutions revealed two characteristic time scales ($\Delta\, <\, 250\,{\mathrm{ms}}$ and $\Delta\, > \, 250\,{\mathrm{ms}}$). As for further analyses we focused on the sharper of those time scales, we re-ran CAD*opti* and the pruning algorithm only for $\Delta \in \{ 0.01,0.015,0.03,0.5,0.8,0.12,0.25\}$. This was done to avoid the loss of those assemblies present at both sharp and broad time scale and assigned by the pruning algorithm to the latter one. In the rest of the paper we focused only on this assembly subset.

### Reinforcement learning model selection

In go/no-go tasks, lick responses can be divided into two components^70^: Initially, upon CS onset, animals display an impulsive Pavlovian lick response that then quickly evolves into an instrumental lick response to CS. We modeled this instrumental lick response as action of a Q-learning model^33^. We considered here three models of action selection generalized from a hybrid Q-learning model with Rescorla–Wagner rule^71^ and Pearce–Hall update mechanism (Q-PH)^36^. In Q-PH models, the value of choosing an action *a* at state *s* is parametrized by the variable *Q*_*s,a*_. The Pearce–Hall update mechanism generalizes the classic Rescorla–Wagner rule by introducing an associability variable *a*, which tracks the uncertainty of the animal on the trial outcome and is proportional to its attention to CS^34,36,72,73^. The associability affects the action value *Q*_*s,a*_ as a feedback-dependent dynamical learning rate. At each trial *t*, *Q*_*s,a*_ is updated according to the following rule:

1 $$Q_{s,a}\left( {t + 1} \right) = Q_{s,a}\left( t \right) + \kappa \alpha (t)\delta (t)$$

where *κ* is a fixed learning parameter and $\delta \left( t \right) = r\left( t \right) - Q_{s,a}\left( t \right)$ is the prediction error, defined as the mismatch between the obtained reward $r\left( t \right)$ and the expected reward $Q_{s,a}\left( t \right)$. The associability update rule depends on the parameter *η* and is

2 $$\alpha \left( {t + 1} \right) = \left( {1 - \eta } \right)\alpha \left( t \right) + \eta \left| {\delta (t)} \right|\eta \in [0,1].$$

In our reinforcement paradigm, mice were presented with one of two odors, states $s \in \{ {\mathrm{geranium}},{\mathrm{ylang}}{\hbox{-}}{\mathrm{ylang}}\}$, and could perform two possible actions $a \in \{ {\mathrm{lick}}\;3\;{\mathrm{times}},{\mathrm{restrain}}\;{\mathrm{licking}}\}$. To better capture our reinforcement learning protocol we compared here three generalizations of the Q-PH model. We considered a: (1) Q-PH model with separate learning rates *κ*_*i*_ for the two actions *a*_*i*_; (2) a Q-PH model with separate parameter *η*_*i*_ for the associability update rule when performed different actions *a*_*i*_; (3) a Q-PH model with forgetting rate *a*_*F*_.

*Q-PH with two learning rates κ (Q-PHκ)*. As shown in by the performance curve of hits and correct rejections (Fig. 3b), mice learned to lick faster than they learn to refrain from licking. To capture this aspect of learning, we generalized the Q-PH model by introducing two distinct learning rates, *κ*_1_ and *κ*_2_, for the two actions *a*_1_ and *a*_2_. Thereby, two update rules were used for the different actions:

3 $$Q_{s,a_1}\left( {t + 1} \right) = Q_{s,a_1}\left( t \right) + \kappa _1\alpha (t)\delta (t)\;\;{\mathrm{when}}\;{\mathrm{mice}}\;{\mathrm{performed}}\;a_1$$

4 $$Q_{s,a_2}\left( {t + 1} \right) = Q_{s,a_2}\left( t \right) + \kappa _2\alpha (t)\delta (t)\;\;{\mathrm{when}}\;{\mathrm{mice}}\;{\mathrm{performed}}\;a_2.$$

*Q-PH with two η (Q-PHη)*. An alternative way to capture the diverse contribution of *a*_1_ and *a*_2_ to learning is to estimate two separate parameters *η*_1_ and *η*_2_ for the associability update rule. This resulted in

5 $$\alpha \left( {t + 1} \right) = \left( {1 - \eta _1} \right)\alpha \left( t \right) + \eta _1\left| {\delta (t)} \right|\eta _1 \in [0,1]\;\;{\mathrm{when}}\;{\mathrm{mice}}\;{\mathrm{performed}}\;a_1$$

and

6 $$\alpha \left( {t + 1} \right) = \left( {1 - \eta _2} \right)\alpha \left( t \right) + \eta _2\left| {\delta (t)} \right|\eta _2 \in [0,1]\;\;{\mathrm{when}}\;{\mathrm{mice}}\;{\mathrm{performed}}\;a_2.$$

*Q-PH with forgetting (Q-PHf)*. While in the two models considered above, only *Q*_*s,a*_(*t*) relative to the performed action is updated, in the Q-PH*f* model we included a forgetting rate *α*_*F*_ to update, at each trial, also the value assigned to the unchosen action *a*′^37^. Thereby, the update rule of *Q*_*s,a*_(*t*) for the chosen action *a* results in

7 $$Q_{s,a}\left( {t + 1} \right) = Q_{s,a}\left( t \right) + \kappa _L\alpha _L(t)\delta (t)$$

8 $$\alpha _L\left( {t + 1} \right) = \left( {1 - \eta } \right)\alpha _L\left( t \right) + \eta \left| {\delta (t)} \right|\eta \in \left[ {0,1} \right],$$

and that of *Q*_*s,a*′_(*t*) for the unchosen action *a*′:

9 $$Q_{s,a^\prime }\left( {t + 1} \right) = Q_{s,a^\prime }\left( t \right) - \kappa _F\alpha _F(t)\;Q_{s,a^\prime }\left( t \right)$$

10 $$\alpha _F\left( {t + 1} \right) = \left( {1 - \eta } \right)\alpha _F\left( t \right) + \eta \;Q_{s,a^\prime }\left( t \right)\eta \in [0,1].$$

For all models, the probability to perform an action *a* at state *s* depends on the action value *Q*_*s,a*_(*t*) according to the equation $p\left( {a{\mathrm{|}}s} \right) = e^{\beta Q_{s,a}(t)}/\mathop {\sum }_l e^{\beta Q_{s,l}(t)}$, where the inverse temperature parameter *β* reflects the exploitation/exploration trade-off. Finally, the model parameters *θ* were estimated from the behavioral data (time series of performed actions in both original and reversal phase) by log-likelihood maximization (using MATLAB’s function *fmincon*, with optimality tolerance = 10^−6^ and 21,296 random initial conditions to avoid local minima). The estimated parameters for the three models were, respectively: $\theta _{QPH\kappa } = \left\{ {\kappa _1,\kappa _2,\eta ,\beta } \right\}$*;* $\theta _{QPH\eta } = \left\{ {\kappa ,\eta _1,\eta _2,\beta } \right\}$*;* $\theta _{fQPH} = \left\{ {\kappa _L,\kappa _F,\eta ,\beta } \right\}$ and all shared the boundary conditions $\eta \in [0,1]$ and $\beta \in [0,500]$. Finally, we used for initialization: $\alpha (1) = 0$ (also for *α*_*L*_ and *α*_*F*_) and $Q_{s,a}(1) = 0.5$ (also for *a*_1_, *a*_2_, and *a*′).

### Reinforcement learning model comparison

Model comparison was performed computing the Bayesian information criterion (BIC). BIC estimates indicated that Q-PH*f* and Q-PHκ outperformed Q-PH*η* in describing behavioral data (Friedman test, main effect: *p* = 7.4 × 10^−20^. Post hoc: Q-PH*f* vs Q-PH*η*: *p* = 1.0 × 10^−9^, Q-PH*κ* vs Q-PH*η*: *p* = 1.0 × 10^−9^). No significant difference was found between the BIC values of Q-PH*f* and Q-PH*κ* (post hoc: Q-PH*f* vs Q-PH*κ*: *p* = 0.2). Since Q-PH*f* had the smallest BIC value and the evidence against the higher BIC was positive according to the Raftery criterion^74^, the model was chosen for further analyses.

Note that the Q-PH*f* becomes the hybrid Rescorla–Wagner model when we set *κ*_*F*_ = 0. The utility of adding an extra parameter was tested with the likelihood ratio test for each animal used for the assembly analysis, and resulted to be significantly in favor of the Q-PH*f* in the 95% of the cases.

### Assembly activity regression on *V*_CS+_

Since the sum of the assembly activity in the 0.7 s after CS+ followed a Poisson distribution, we evaluated the correlation between the subjective value assigned to CS+ at each trial and SPN–DAN assembly activity using a Poisson regression model. The Poisson regression was defined by the equation $\log \left( {\mu _t} \right) = \beta _0 + \beta \cdot V_{{\mathrm{CS}} + }\left( t \right)$, where *μ*_*t*_ is the expected value of the summed assembly activity, *β* is the regression coefficient, and *V*_CS+_(*t*) is the value assigned to a specific state *s*. At each trial, *V*_CS+_ is extracted from the action values *Q* of the Q-PH*f* model according to $V_{{\mathrm{cs}} + }\left( t \right) \equiv \mathop {{\max }}\limits_a \left( {Q_{{\mathrm{cs}} + ,a}\left( t \right)} \right)$. To pool and compare different assemblies and different sessions, the regression coefficients were standardized as $\bar \beta = \beta \cdot \sigma (V)/\sigma (\mu )$ and further transformed as $\beta ^\ast = {\mathrm{exp}}\left( {\bar \beta } \right) - 1$, to improve interpretability. In this way, positive β* indicated positive correlations between assembly activity and *V*_CS+_, and vice versa. In Fig. 5d, we reported only those β* significantly different from 0 (*t*-statistics).

To exclude that the obtained result could depend on drifts in assembly baseline activity, we subtracted the baseline activity from the CS+ triggered activity of each assembly and repeated the analysis^75^. Assembly activity continued to be significantly correlated with *V*_CS+_. All significant β* were positive.

### Reporting summary

Further information on research design is available in the Nature Research Reporting Summary linked to this article.

## Supplementary information

Supplementary Information

Reporting Summary

## Data Availability

All data reported in this study are available from the corresponding authors upon request. The source data underlying Figs. 1c, d, h, 2e, 3c, d, 4b, d, 5c, d and Supplementary Figs. 2d, f, 3e, f, 5a, b, 7d, h, I, 8c, 9d–f and 10a, e, f–h are provided as a Source data file. A reporting summary for this Article is available as a Supplementary Information file. Source data are provided with this paper.

## Source data

Source Data

## Code availability

Code for cell assembly detection at multiple time scales (CAD*opti*) available at https://github.com/DurstewitzLab/CADopti. Source data are provided with this paper.
