# Supplementary material for: Phasic dopamine reinforces distinct striatal stimulus encoding in the olfactory tubercle driving dopaminergic reward prediction
Source: Nat Commun. 2020 Jul 10;11:3460. doi: 10.1038/s41467-020-17257-7 (PMC7351739; doi:10.1038/s41467-020-17257-7)
Supplement: Supplementary file 1 — Supplementary Information [file 41467_2020_17257_MOESM1_ESM.pdf]

Supplementary Information for

Phasic dopamine reinforces distinct striatal stimulus encoding in the olfactory tubercle driving dopaminergic reward prediction

Oettl et al.

Includes:

Supplementary Figures 1-11

SUPPLEMENTARY FIGURES

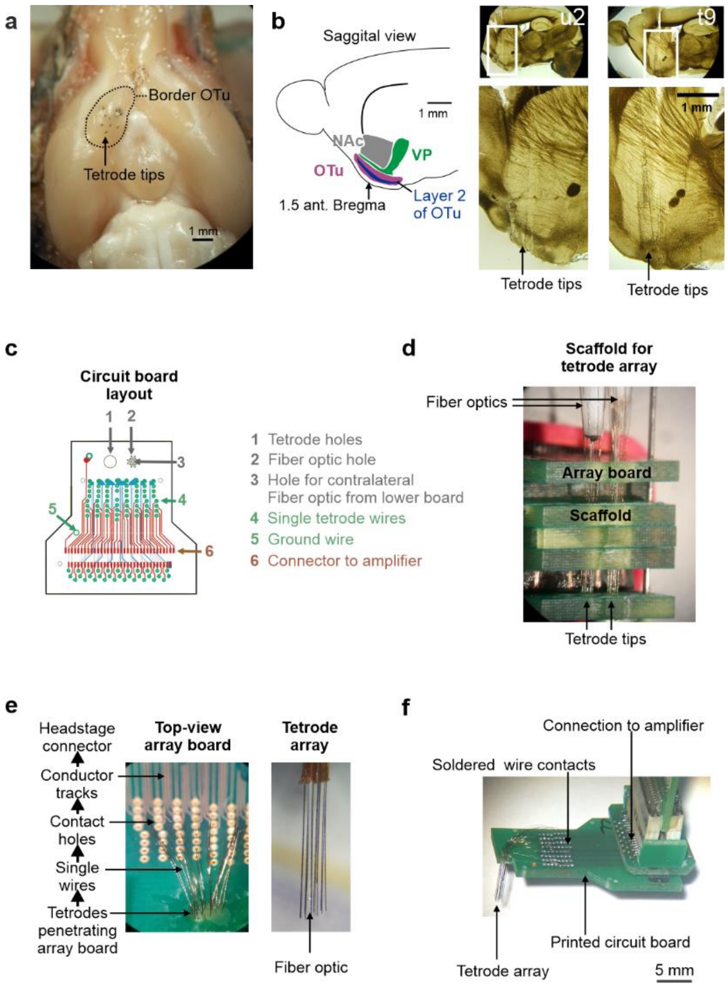

Supplementary Figure 1. Recordings of SPN in the olfactory tubercle of ventral striatum. Related to Figure 1. **a**, Exemplary histological confirmation of the placement of the tetrodes in the olfactory tubercle (OTu) in a ventral forebrain view. **b**, Scheme with the anatomical relation of ventral striatal brain regions in sagittal view and exemplary histological confirmation of the placement of the tetrodes in the OTu in sagittal sections from two mice #u2 and #t9. **c-f**, Illustration of the building of the tetrode array. (c) Exemplary layout of the printed circuit board. (d) A scaffold was used to arrange tetrodes in parallel. (e) Single wires of tetrodes were connected to the board and fixed by soldering. Example of a tetrode array with a fiber optic in the center. (f) The array connected to the breakout board of the head stage connector.

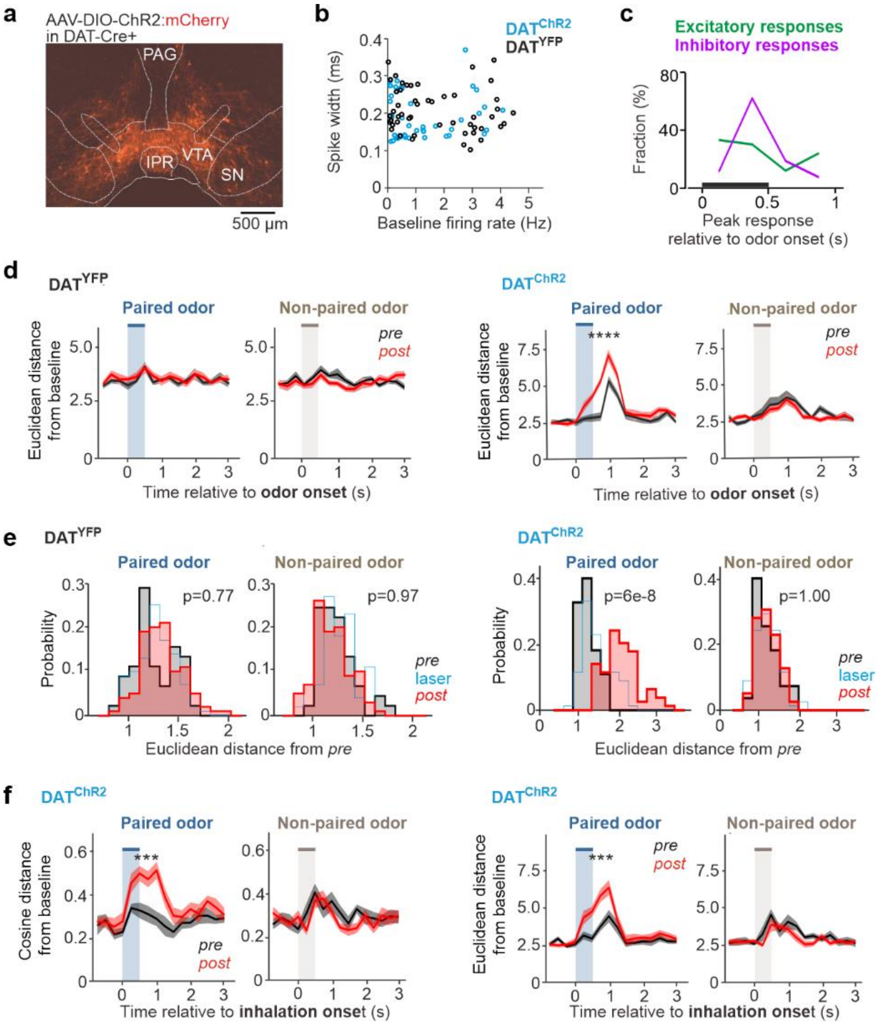

Supplementary Figure 2. Phasic DA modifies the striatal population code of the paired odor selectively. Related to Figure 1. **a**, Conditional expression of ChR2:mCherry in the VTA of a DAT-Cre mouse from the recordings in Fig. 1 upon virus injection of AAV-DIOA-ChR2:mCherry after completion of the recordings in a coronal section (Bregma: -3.2 mm). Similar results were obtained from all 6 DAT^{ChR2} mice. Abbreviations: IPR, interpeduncular nucleus (rostral); PAG, periaqueductal gray; SN, substantia nigra. **b**, The baseline firing rate was plotted against the width at half maximum of the median spike waveform of SPN from DAT^{ChR2} (blue) and DAT^{YFP} (black) mice. **c**, Time to peak change in firing rate during the odor response was plotted for all excitatory and inhibitory responses pooled from DAT^{ChR2} and DAT^{YFP} mice (250 ms bins) (same response classification as in Fig. 1h and Supplementary Fig. 3e-f). **d**, Same as Figure 1c-d but computed with the Euclidean metric. DAT^{YFP}: n=10 trial-averages of 3 trials respectively for pre and post, respectively. DAT^{ChR2}: n=8 and n=10 trial-averages for 3 trials for pre and post. Data displayed as mean ± S.E. **e**, Same as Figure 1e-f respectively but computed with the Euclidean metric. Significance established with a three-way ANOVA (factors: cohort, phase, and odor). Interaction effect: $F(1,498)=40.3$; $p=5 \times 10^{-10}$. Post-hoc tests (Tukey's correction) are reported on the plots. **f**, Same as Figure 1c-d, but computed for neuronal activity aligned to the first inhalation after stimulus onset. Source data are provided as a Source Data file.

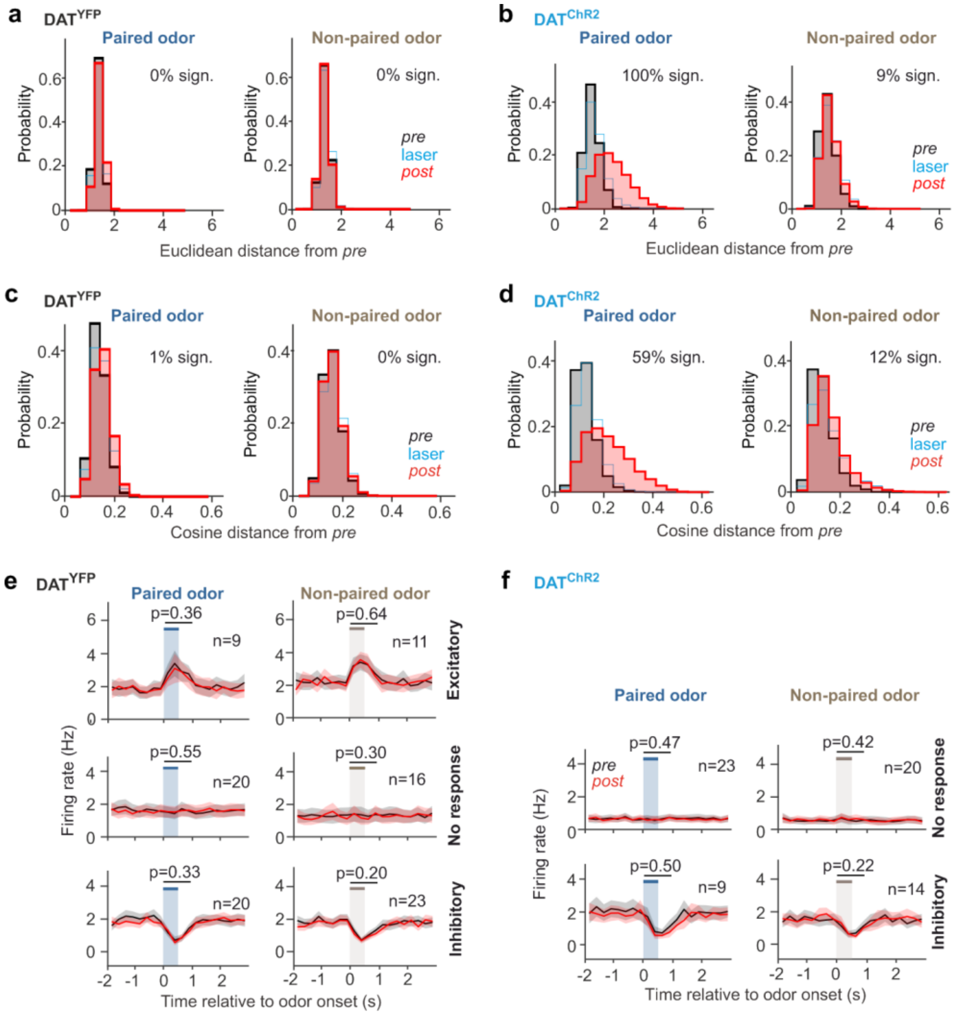

Supplementary Figure 3. Phasic DA modifies the striatal population code selectively of the paired odor. Related to Figure 1. **a-b**, For the data in Figure 1e-f, the analyses were repeated 300 times with random permutation, at each repetition, of the order of trial pairing across sessions used to build the population vectors with Euclidean metric. For each repetition we performed a three-way ANOVA (factors: cohort, phase, and odor). (b) Of the 300 repetitions the interaction effect between the three factors was found significant 100% of the times in DAT^{ChR2} mice. The fraction of significant post-hoc comparisons (Tukey's correction) is indicated. **c-d**, same as (a-b) respectively, but with distances computed using the cosine metric. (d) Of the 300 repetitions the interaction effect between the three factors was found significant 59% of the times in DAT^{ChR2} mice. The fraction of significant post-hoc comparisons (Tukey's correction) is indicated. **e**, Mean PSTH ± S.E. of SPN with excitatory (top), no (middle), and inhibitory responses (bottom) to the paired (left) and non-paired odor in DAT^{YFP} mice (two-sided paired Wilcoxon signed rank test on the averaged rate from 0 to 1 s). **f**, same as (e) for DAT^{ChR2} mice (two-sided paired Wilcoxon signed rank test on the averaged rate from 0 to 1 s). The excitatory responses are shown in Figure 1h. Source data are provided as a Source Data file.

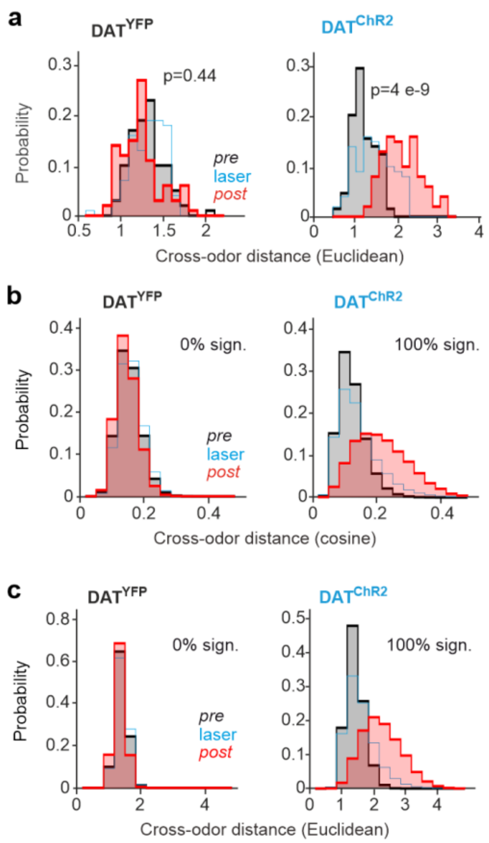

Supplementary Figure 4. Phasic DA increases the difference between paired and non-paired odor representations. Related to Figure 2a. **a**, Same as Figure 2a but with the use of the Euclidean metric. Statistical test: two-way ANOVA (factors: cohort and odor). Interaction effect: $F(1,360)=227.5$ $p=4 \times 10^{-40}$, post-hoc comparisons (Tukey's correction) reported on the plots. **b-c**, The analyses performed respectively for (a) and Figure 2a were repeated 300 times with random permutations, at each repetition, of the order of trial pairing across-sessions used to build the population vectors. In DAT^{ChR2} mice, the percentage of tests with significant interaction effect was 100% both for the cosine (b) and the Euclidian metric (c). Fraction of significant post-hoc comparisons (Tukey's correction) indicated on the Figure.

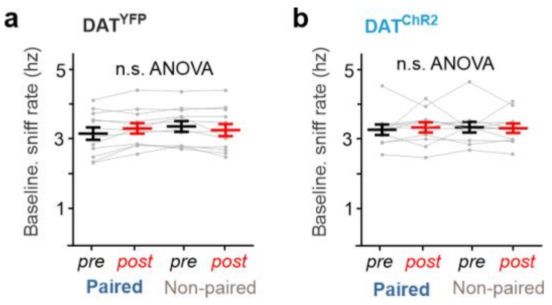

Supplementary Figure 5. Phasic DA increases persistently the perceived salience of the paired odor. Related to Figure 2e. **a**, Mean sniff rate ± S.E. directly before the odor application (baseline rate). Data are plotted as mean across DAT^{YFP} animals before and after the pairing protocol (one-way ANOVA $F(3,44)=0.28$; $p=0.84$). **b**, Same as (a), but for DAT^{ChR2} mice (one-way ANOVA $F(3,40)=0.05$; $p=0.98$). DAT^{YFP}: n=12 sessions. DAT^{ChR2}: n=11 sessions. Source data are provided as a Source Data file.

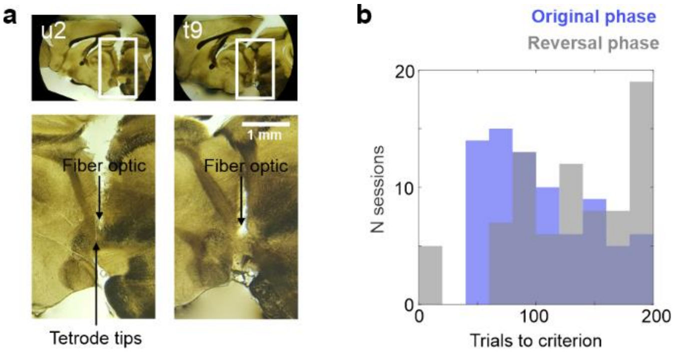

Supplementary Figure 6. Placement of recording array in VTA and trials to criterion in the reversal learning task. Related to Figure 3. **a,** Exemplary histology to confirm the placement of the tetrodes and fiber optics in the VTA in sagittal sections from two mice of the cohort (same mice as shown in Figure S1b). **b,** Number of trials needed to reach criterion performance (80% accuracy in 50 consecutive trials) for the original and reversal phase in all sessions used in the analyses.

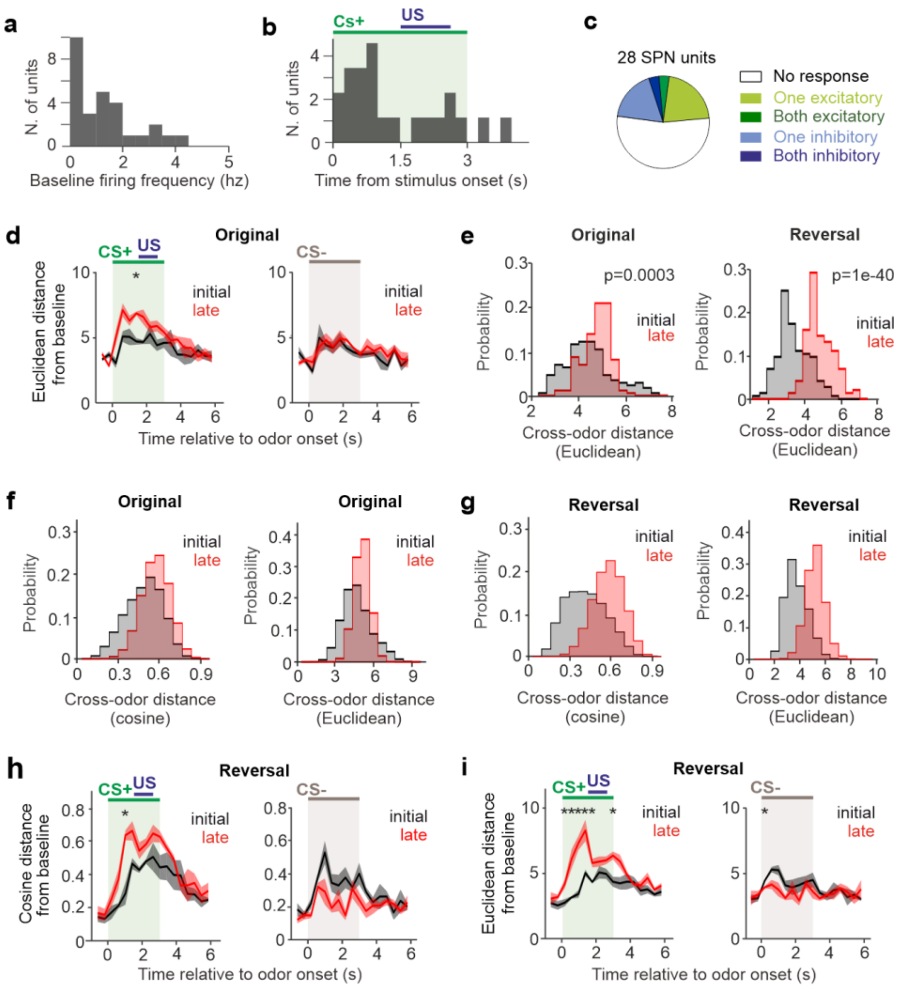

Supplementary Figure 7. Stimulus related network representations are modified during reversal learning. Related to Figure 3. **a**, Baseline firing rates of the SPN units in the sample in Fig. 3. **b**, Time from odor onset to the peak z-score of the response of SPN units in Fig. 3. **c**, Fraction of SPN response types to the stimuli in OTu in Fig. 3. **d**, Mean temporal evolution ± S.E. of the Euclidian distance of the population vector from baseline for the rewarded and non-rewarded odor for the 'initial' and 'late' trials. Significant changes between the 'initial' and 'late' trials were assessed with a two-tailed t-test corrected for multiple comparisons across bins (asterisk indicates significance). During learning the population vector for CS+, but not for CS-, significantly increased its distance from baseline. DAT^{YFP}: n=4 trial-averages of 3 trials respectively for initial and late. **e**, Distribution of Euclidian distances between the trial-specific neuronal representations of CS+ and CS- within all 'initial' trials and all 'late' trials. Significance was assessed with a two-tailed t-test. During learning the population vectors relative CS+ and CS- diverged. Left: original phase. Right: reversal phase. **f-g**, To exclude that the results obtained in Fig. 3f and Supplementary Fig. 7e depend on a specific trial-alignment in the construction of the population vectors from multiple sessions, we repeated the analyses 300 times on population vectors obtained by randomly permuting the order of trial pairing across animals. In 100% of the 300 repetitions the distance between the two odor responses (computed either with the cosine or with the Euclidean metric) increased with learning. The effect was present both during the (f) original and (g) reversal phase. **h**, same as Fig. 3c, but cosine distance to baseline computed on the trials following the reversal of the odor-reward pairing. **i**, same as (d), but Euclidian distance to baseline computed on the trials following the reversal of the odor-reward pairing. Source data are provided as a Source Data file.

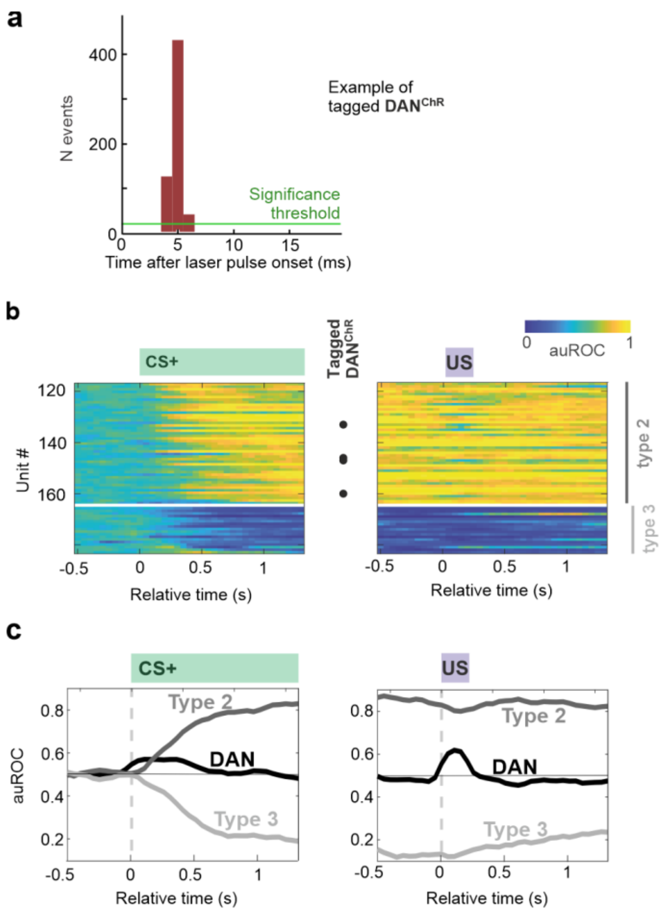

Supplementary Figure 8. Optogenetic tagging of DAN. Related to Figure 4a. **a**, Example of an optogenetically tagged DAN. Histogram (1 ms bins) of spikes after laser pulses. Green line represents computed significance threshold for optogenetic activation (see Methods: Optogenetic identification of DAN). **b**, Classification of VTA units by functional clustering. Response traces as in Fig. 4a. Traces were hierarchically clustered into three groups: putative DAN (type 1), GABAergic interneurons (type 2) and glutamatergic interneurons (type 3). Here displayed only type 2 and type 3 units. DAN are shown in Fig. 4a. **c**, The mean auROC response is shown for the three cell-types from (b) and Fig. 4a. Source data are provided as a Source Data file.

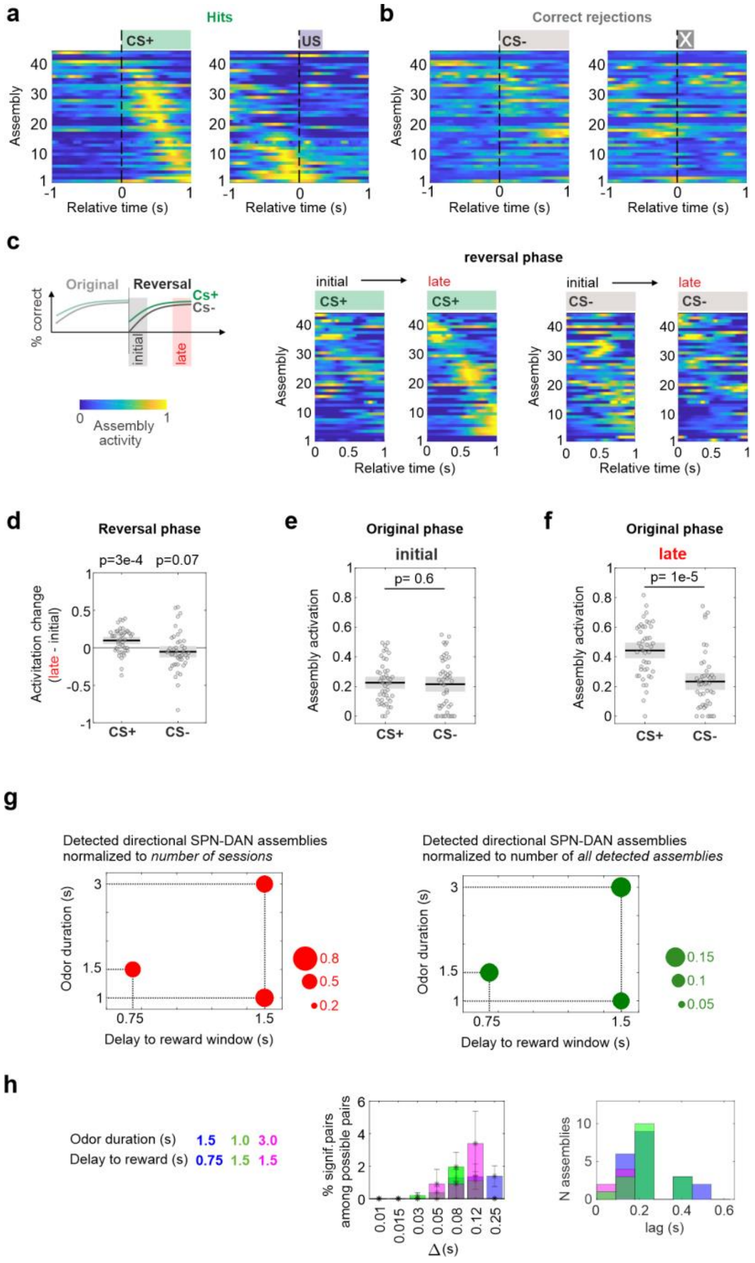

Supplementary Figure 9. SPN-DAN assemblies emerge with learning. Related to Figure 5. **a-b**, Assembly activity of SPN-DAN assemblies averaged across (a) hit trials and (b) correct rejection trials of the original phase. SPN-DAN assembly activation during CS+ was higher than during CS- (two-sided Wilcoxon test, $p=7.55 \times 10^{-6}$). Throughout figure: CS window = 0-0.7 s from CS onset. **c**, same as in Fig. 5a-b for the reversal phase. **d**, same as in Fig. 5c for the reversal phase. The difference was significant for CS+ but not CS- (Wilcoxon test). n=45 assemblies. **e-f**, Average activity of directional assemblies at CS during the (e) initial and (f) late trials of the original phase. Mean ± S.E. responses to CS+ differed from CS- only in the late trials, in agreement with the formation of the odor-outcome association (two-sided Wilcoxon test). n=45 assemblies. **g**, Relative occurrence of directional SPN-DAN (left) normalized to the number of sessions with different lengths of the odor presentation and delays from odor onset to the onset of the retrieval window. (Right) Same as (left) with normalization to the total number of inter-regional assemblies of any cell-type. Directional SPN-DAN assemblies occurred robustly independently of specific paradigm settings. **h**, Temporal precision Δ and lags of assemblies plotted divided by the different lengths of the odor presentation and delays from odor onset to the onset of the retrieval window. Data displayed as mean ± S.E., n=8/5/2 for the blue/green/pink data sets, respectively. Source data are provided as a Source Data file.

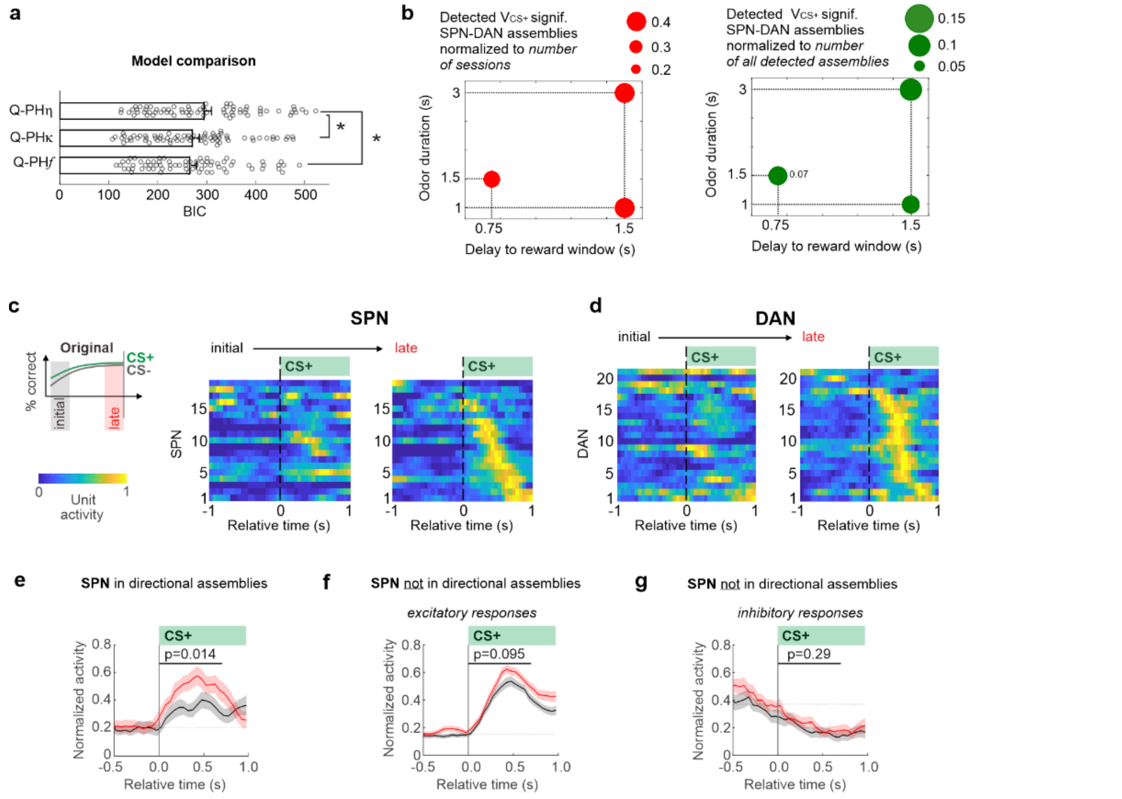

Supplementary Figure 10. Reinforcement learning in SPN-DAN assemblies. Related to Figure 5. **a**, Mean Bayesian Information Criterion (BIC) over all animals for the three presented models. The Q-PH*f* model showed the lowest BIC and was selected for further analyses (Friedman test, main effect: $p=7.4 \times 10^{-20}$. post-hoc: Q-PHf vs Q-PHη: $p=1.0 \times 10^{-9}$, Q-PHκ vs Q-PHη: $p=1.0 \times 10^{-9}$. Each model: n=69 samples. Data displayed as mean ± S.E.). **b**, Relative occurrence of directional SPN-DAN assemblies significantly correlated with V_{CS+} (left) normalized to the number of sessions with different lengths of the odor presentation and delays from odor onset to the onset of the retrieval window. (Right) Same as (left) with normalization to the total number of inter-regional assemblies of any cell-type. Directional SPN-DAN assemblies significantly correlated with V_{CS+} occurred robustly independently of specific paradigms. **c-d**, Activity of (c) SPN and (d) DAN from assemblies positively correlated with V_{CS+}. The response to CS+ increased in both SPN and DAN during stimulus-outcome learning (one-tailed Wilcoxon test: p=0.008 for SPN, p=0.01 for DAN), demonstrating that the assembly modulation observed on Fig. 5c-d is not exclusively driven by changes in either one of the two cell types only. CS window = 0-0.7 s from CS onset. **e-g**, Plot of the mean ± S.E. normalized firing rate in response to CS+ of SPN (e) participating in SPN-DAN directional assemblies (n=19) or (f-g) not participating in these assemblies. The latter are separated in SPN with (f) excitatory or (g) inhibitory responses (n=70 or 26, respectively) to CS+. Two-tailed Wilcoxon signed rank test of the averaged normalized activity during 0 to 0.7 s relative to odor onset. Source data are provided as a Source Data file.

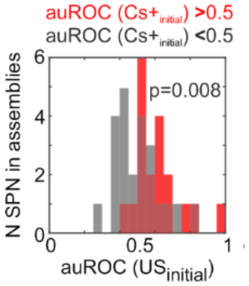

Supplementary Figure 11. Shared SPN responses at CS+ and US. Related to Figure 5. Distributions of $auROC(US_{initial})$ for units with enhanced ($auROC(CS+_{initial})$>0.5) or reduced ($auROC(CS+_{initial})$<0.5) responsiveness to CS+. The $auROC(CS+_{initial})$ compared the distribution of spike counts at CS+ to baseline in the initial trials. SPN with an excitatory response to CS+ in the initial trials show preferentially also an excitatory response to US (two-sided Wilcoxon test).
