# Supplementary material for: Phasic dopamine reinforces distinct striatal stimulus encoding in the olfactory tubercle driving dopaminergic reward prediction
Source: Nat Commun. 2020 Jul 10;11:3460. doi: 10.1038/s41467-020-17257-7 (PMC7351739; doi:10.1038/s41467-020-17257-7)
Supplement: Supplementary file 2 — Reporting Summary [file 41467_2020_17257_MOESM2_ESM.pdf]

nature research

Corresponding author(s): Wolfgang Kelsch

Last updated by author(s): Apr 11, 2020

Reporting Summary

Nature Research wishes to improve the reproducibility of the work that we publish. This form provides structure for consistency and transparency in reporting. For further information on Nature Research policies, see Authors & Referees and the Editorial Policy Checklist.

Statistics

For all statistical analyses, confirm that the following items are present in the figure legend, table legend, main text, or Methods section.

n/a	Confirmed	
☐	☒	The exact sample size (*n*) for each experimental group/condition, given as a discrete number and unit of measurement
☐	☒	A statement on whether measurements were taken from distinct samples or whether the same sample was measured repeatedly
☐	☒	The statistical test(s) used AND whether they are one- or two-sided<br>*Only common tests should be described solely by name; describe more complex techniques in the Methods section.*
☐	☒	A description of all covariates tested
☐	☒	A description of any assumptions or corrections, such as tests of normality and adjustment for multiple comparisons
☐	☒	A full description of the statistical parameters including central tendency (e.g. means) or other basic estimates (e.g. regression coefficient) AND variation (e.g. standard deviation) or associated estimates of uncertainty (e.g. confidence intervals)
☐	☒	For null hypothesis testing, the test statistic (e.g. *F*, *t*, *r*) with confidence intervals, effect sizes, degrees of freedom and *P* value noted<br>*Give P values as exact values whenever suitable.*
☒	☐	For Bayesian analysis, information on the choice of priors and Markov chain Monte Carlo settings
☐	☒	For hierarchical and complex designs, identification of the appropriate level for tests and full reporting of outcomes
☐	☒	Estimates of effect sizes (e.g. Cohen's *d*, Pearson's *r*), indicating how they were calculated

Our web collection on statistics for biologists contains articles on many of the points above.

Software and code

Policy information about availability of computer code

Data collection	Matlab, Intan Tech GUI
Data analysis	Matlab, Graphpad Prism

For manuscripts utilizing custom algorithms or software that are central to the research but not yet described in published literature, software must be made available to editors/reviewers. We strongly encourage code deposition in a community repository (e.g. GitHub). See the Nature Research guidelines for submitting code & software for further information.

Data

Policy information about availability of data

All manuscripts must include a data availability statement. This statement should provide the following information, where applicable:

- Accession codes, unique identifiers, or web links for publicly available datasets
- A list of figures that have associated raw data
- A description of any restrictions on data availability

The data that support the findings of this study are available from the corresponding author upon request.

Field-specific reporting

Please select the one below that is the best fit for your research. If you are not sure, read the appropriate sections before making your selection.

☒ Life sciences ☐ Behavioural & social sciences ☐ Ecological, evolutionary & environmental sciences

For a reference copy of the document with all sections, see nature.com/documents/nr-reporting-summary-flat.pdf

Life sciences study design

All studies must disclose on these points even when the disclosure is negative.

Sample size	Sample sizes (number of single units within a single analyses and number of animals per group) were chosen according to previously used numbers in own awake recordings and comparable studies published from other labs sufficient to detect medium or large size effects (eg. Broome et al. (2006) Neuron 51: 467-482, Yamada et al. (2017) Neuron 93, 1198–1212). Since the exact variability of the effects was unknown, sample size calculations were not reliably possible.
Data exclusions	Experimental cohorts were completed and single units extracted. Only behavioral sessions were included in which the animal reached criterion (80% correct (hit and rejection) trials). Units were only included in the further analyses if they fulfilled predetermined criteria for single units: All units with a firing rate < 2 Hz were classified as SPN. All units with a firing rate > 12 Hz were excluded as putative fast spiking neurons from the OTu or from the directly neighboring ventral pallidum. Units in the ambiguous range from 2-12 Hz were designated as putative regular-firing cholinergic interneurons if the coefficient of variation of their inter-spike interval (ISI) distribution was less than 1.2 and ISIs of less than 60 ms contributed no more than 20% of all ISIs . The remaining units were assigned as SPN if they ever paused firing for more than 2 s (with a fraction of ISIs bigger than 2s > 1/10-4). Neurons that did not fulfill this latter criterion were considered fast spiking neurons.<br><br>Additionally, in the evoked phasic DA experiment (Fig. 1,2), as pre-established criterion, single units were excluded if their baseline firing rate changed by more than 10% throughout the course of the experiment. This step was part of the workflow as we analyzed changes relative to baseline at the population level and these analyses are highly sensitive to such baseline drifts. No further data exclusion was applied.
Replication	The study examined a novel phenomenon. The study design did not contain generally full replication cohorts. The experiments involving reversal learning were however designed to test whether the emergence of interregional assemblies encoding reward prediction can be replicated in different experiments varying the settings (i.e. different odor presentation duration and time from odor onset to reward window). All attempts at replication were successful. The results are shown respectively in the Extended Data Figures S9g and S10b.
Randomization	Mouse cohorts within one experiment were of the same sex, genotype and comparable age (ca. 12 weeks at the beginning of the experiment), but received two different viruses (expressing either ChR2:mCherry or GFP control). All animals of the dopamine induced plasticity underwent the same experimental procedure. The reversal learning experiment only contained one group of mice that all underwent the same training and testing. Therefore, in both experiments, no further randomizations were necessary at the subject level.<br><br>Considering the relatively small cell count per session due to the numerous selection criteria (see Main manuscript), we built spike count population vectors by pooling units from different sessions as established broadly for in vivo recordings (cf. Ref. 12–14 in Extended data). We excluded that the specific order of trial concatenation across sessions influenced our results by repeating all main analyses with 300 random permutations of cross-session trial matching (for further details please see Method section: 'Population analysis: The population vector.' and Extended Data Figure S3a-d and S4b-c).
Blinding	In the reversal learning experiment, blinding was not relevant as all animals received the same treatment (training, task performance and analyses). In the dopamine plasticity experiment, experimenters were single blinded during spike detection for ChR2:mCherry or GFP control expression, then the experimenter was unblinded to assign single units to ChR2:mCherry or GFP control expression groups. The further analyses on the ChR2 and control group was performed with same scripts.

Reporting for specific materials, systems and methods

We require information from authors about some types of materials, experimental systems and methods used in many studies. Here, indicate whether each material, system or method listed is relevant to your study. If you are not sure if a list item applies to your research, read the appropriate section before selecting a response.

Materials & experimental systems

n/a	Involved in the study	
☒	☐	Antibodies
☐	☒	Eukaryotic cell lines
☒	☐	Palaeontology
☐	☒	Animals and other organisms
☒	☐	Human research participants
☒	☐	Clinical data

Methods

n/a	Involved in the study	
☒	☐	ChIP-seq
☒	☐	Flow cytometry
☒	☐	MRI-based neuroimaging

Eukaryotic cell lines

Policy information about cell lines

Cell line source(s)	CRL-11268 293T/17 Embryonic Kidney Human directly obtained from ATCC (LOT:62312975)
Authentication	Cell line was directly obtained from ATCC LOT:62312975. The cell line used was not further authenticated.

Mycoplasma contamination	Not tested since cell line is only used with low passages in the lab (<20) and for production of recombinant AAV for gene delivery in mice
Commonly misidentified lines (See ICLAC register)	No commonly misidentified cell lines were used.

Animals and other organisms

Policy information about studies involving animals; ARRIVE guidelines recommended for reporting animal research

Laboratory animals	Directly obtained from Jackson Labratory and were maintained in a heterozygous C57BL/6J (Charles-River):<br>1. DAT:(IRES)Cre mice (B6.SJL-Slc6a3tm1.1(cre)Bkmn/J; RRID: IMSR_JAX:006660<br>2. B6;129S-Gt(ROSA)26Sortm32(CAG-COP4*H134R/EYFP)Hze/J)<br><br>Mice were single housed upon implantation of the recording array at a 12 hours' day-and-night-cycle (room temperature approx. 24°C, air humidity 45 to 50%). Recordings were performed with male mice that were 3 to 6 months old.
Wild animals	No wild animals were used in the study.
Field-collected samples	No field collected samples were used in the study.
Ethics oversight	Regierungspräsidium Karlsruhe, State of Baden-Wuerttemberg, Germany

Note that full information on the approval of the study protocol must also be provided in the manuscript.
